# Evolution and neutralization escape of the SARS-CoV-2 BA.2.86 subvariant

**DOI:** 10.1038/s41467-023-43703-3

**Published:** 2023-12-06

**Authors:** Khadija Khan, Gila Lustig, Cornelius Römer, Kajal Reedoy, Zesuliwe Jule, Farina Karim, Yashica Ganga, Mallory Bernstein, Zainab Baig, Laurelle Jackson, Boitshoko Mahlangu, Anele Mnguni, Ayanda Nzimande, Nadine Stock, Dikeledi Kekana, Buhle Ntozini, Cindy van Deventer, Terry Marshall, Nithendra Manickchund, Bernadett I. Gosnell, Richard J. Lessells, Quarraisha Abdool Karim, Salim S. Abdool Karim, Mahomed-Yunus S. Moosa, Tulio de Oliveira, Anne von Gottberg, Nicole Wolter, Richard A. Neher, Alex Sigal

**Affiliations:** 1 Africa Health Research Institute, Durban, South Africa; 2 School of Laboratory Medicine and Medical Sciences, University of KwaZulu-Natal, Durban, South Africa; 3 Centre for the AIDS Programme of Research in South Africa, Durban, South Africa; 4 Biozentrum, University of Basel, Basel, Switzerland; 5 Swiss Institute of Bioinformatics, Lausanne, Switzerland; 6 Centre for Respiratory Diseases and Meningitis, National Institute for Communicable Diseases, a Division of the National Health Laboratory Service, Johannesburg, South Africa; 7 Ampath Molecular Biology, Centurion, South Africa; 8 Department of Infectious Diseases, Nelson R. Mandela School of Clinical Medicine, University of KwaZulu-Natal, Durban, South Africa; 9 KwaZulu-Natal Research Innovation and Sequencing Platform, Durban, South Africa; 10 Department of Epidemiology, Mailman School of Public Health, Columbia University, New York, NY USA; 11 Centre for Epidemic Response and Innovation, School of Data Science and Computational Thinking, Stellenbosch University, Stellenbosch, South Africa; 12 Department of Global Health, University of Washington, Seattle, WA USA; 13 School of Pathology, Faculty of Health Sciences, University of the Witwatersrand, Johannesburg, South Africa

**Keywords:** Phylogenetics, SARS-CoV-2, Viral evolution

## Abstract

Omicron BA.2.86 subvariant differs from Omicron BA.2 as well as recently circulating variants by over 30 mutations in the spike protein alone. Here we report on the isolation of the live BA.2.86 subvariant from a diagnostic swab collected in South Africa which we tested for escape from neutralizing antibodies and viral replication properties in cell culture. We found that BA.2.86 does not have significantly more escape relative to Omicron XBB.1.5 from neutralizing immunity elicited by either Omicron XBB-family subvariant infection or from residual neutralizing immunity of recently collected sera from the South African population. BA.2.86 does have extensive escape relative to ancestral virus with the D614G substitution (B.1 lineage) when neutralized by sera from pre-Omicron vaccinated individuals and relative to Omicron BA.1 when neutralized by sera from Omicron BA.1 infected individuals. BA.2.86 and XBB.1.5 show similar viral infection dynamics in the VeroE6-TMPRSS2 and H1299-ACE2 cell lines. We also investigate the relationship of BA.2.86 to BA.2 sequences. The closest BA.2 sequences are BA.2 samples from Southern Africa circulating in early 2022. Similarly, many basal BA.2.86 sequences were sampled in Southern Africa. This suggests that BA.2.86 potentially evolved in this region, and that unobserved evolution led to escape from neutralizing antibodies similar in scale to recently circulating strains of SARS-CoV-2.

## Abstract (web summary)

The Omicron BA.2.86 subvariant differs from previous variants by over 30 spike mutations. Here, the authors report that BA.2.86 likely evolved in Southern Africa and that its immune escape is not larger than recently circulating SARS-CoV-2 strains. Neither its replication nor its pathogenicity are enhanced in vitro.

## Introduction

**Fig. 1:**
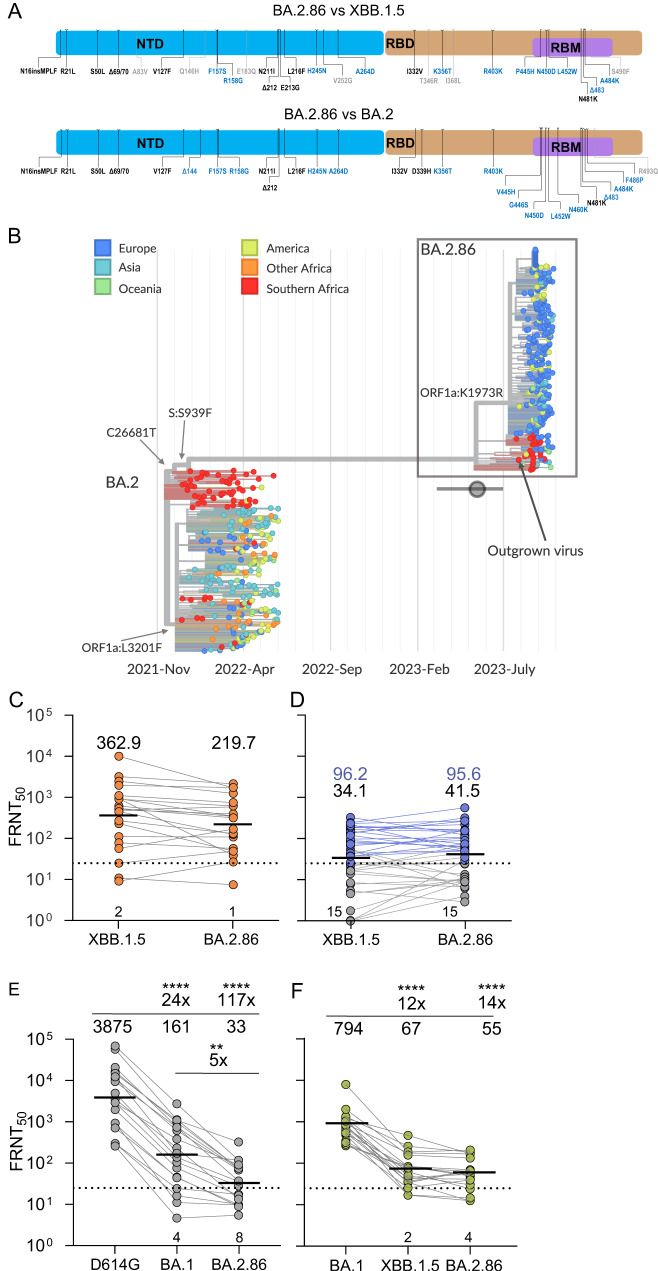
Omicron BA.2.86 evolution and neutralization escape. **A** Changes in BA.2.86 spike relative to Omicron XBB.1.5 and BA.2. Blue shading denotes spike N-terminal domain (NTD), brown is receptor-binding domain (RBD) and purple is the receptor-binding motif (RBM) within the RBD. **B** Phylogenetic analysis. BA.2.86 sequences form a distinct cluster separated from BA.2 sequences circulating in late 2021/early 2022 by a long branch. Outgrown sample marked by the arrow. The BA.2.86 branch connects to samples with the mutations C26681T and C24378T (Spike: S939F) but lacks C9866T (ORF1a:K1973R) present in most BA.2 sequences. The emergence date of BA.2.86 and its uncertainty is indicated by a circle with a horizontal bar. **C** Neutralization of BA.2.86 live virus vs. XBB.1.5 by *n* = 21 sera from individuals with XBB-derived subvariant infection. **D** Neutralization of BA.2.86 vs. XBB.1.5 by *n* = 40 sera from individuals sampled in September 2023. **E** Neutralization of BA.2.86 vs. ancestral D614G and BA.1 viruses by *n* = 19 sera from vaccinated individuals collected before Omicron emergence. **F** Neutralization of BA.2.86 vs. Omicron BA.1 by *n* = 19 sera from Omicron BA.1 infected individuals. For **C**–**F**, the numbers in black above columns are GMT FRNT_50_ for each group and horizontal dashed lines represent the most concentrated plasma used (1:25), corresponding to the limit of quantification (LOQ) of FRNT_50_ = 25, below which values are extrapolated. The number of samples below LOQ is shown just above the *x*-axis. For **D**, gray lines and points are values below, and blue points and lines are above LOQ and numbers in blue above columns are GMT FRNT_50_ for samples above LOQ. For **E** and **F**, significant fold changes are shown. Significant *p*-values for **E** were: ****p = 9 × 10^−6^ (D614G vs. BA.1), ****p = 4.0 × 10^−10^ (D614G vs. BA.2.86), **p = 0.004 (BA.1 vs. BA.2.86). For **F**: ****p = 6 × 10^−10^ (BA.1 vs. XBB.1.5) and ****p = 6 × 10^–11^ (BA.1 vs. BA.2.86). All *p*-values by a two-sided Wilcoxon Rank Sum test.

The Omicron subvariant BA.2.86 is derived from the BA.2 subvariant but has over 30 mutations in spike relative to both BA.2 and recently circulating subvariants such as XBB.1.5 (Fig. 1A), making its emergence a major concern since many of the mutations are predicted to confer escape from neutralizing antibodies^1^.

Based on vaccine efficacy studies^2–14^, levels of neutralizing antibodies have been found to correlate strongly with protection from symptomatic infection with SARS-CoV-2^15^. Mutations occurring in the receptor-binding domain and N terminal domain of spike tend to reduce the ability of antibodies elicited by previous infection or vaccination to neutralize SARS-CoV-2^16–19^. Protection from severe disease requires lower antibody levels^20^, and also involves T cell responses^21–23^, which are mostly conserved despite changes in the spike protein that characterize SARS-CoV-2 variants and subvariants^24,25^.

The Omicron BA.2.86 subvariant started to be identified by global genomic surveillance samples collected from 24 July 2023 onwards, but because of the reduced rate of surveillance the exact time when it started to spread is unclear. Likewise, it is unclear when and where it arose. In this work, we use phylogenetic analysis to investigate the origin of BA.2.86 and neutralization and virological assays to measure escape from neutralizing antibodies and other viral properties to give an indication of how different this variant is likely to be from recently circulating subvariants descended from XBB. We find that BA.2.86 likely evolved in Southern Africa. Despite the extensive genomic changes relative to other circulating variants, our data supports the notion that BA.2.86 is not substantially different from recent variants in escape from neutralizing antibodies or cellular infection.

## Results

BA.2.86 shares the synonymous mutation C26681T and the spike substitution S939F with BA.2 genomes sampled in South Africa in early 2022, while it lacks the mutation C9866T (ORF1a:L3201F) that is present in the great majority of BA.2 sequences sampled outside of Southern Africa (Fig. 1B). Southern African sequences are also closely related to the putative ancestral sequence of BA.2.86. Most samples collected between mid-August and mid-September have 3–7 mutations relative to the most recent common ancestor (MRCA) of BA.2.86 (Fig. S1). Of the 15 branches that emanate from the basal polytomy within BA.2.86, 11 are dominated by samples from Southern Africa (Fig. 1B and Fig. S1). Most sequences from the Northern Hemisphere fall into a second large polytomy designated as BA.2.86.1, separated from the basal polytomy by two mutations, including ORF1a:K1973R (Fig. 1B). This sub-lineage has not been observed in South Africa at the time of analysis. SARS-CoV-2 accumulates about 15 mutations per year along acute transmission chains and we thus estimate that this subvariant started to spread about May 2023^26^. The estimate is corroborated by molecular clock analysis with TreeTime, which suggests an emergence date in mid-May with an uncertainty from early March to early July (Fig. 1B).

The virus isolate tested here is from a nasopharyngeal swab sample collected in Mpumalanga Province, South Africa on July 28, 2023 (Fig. 1B, arrow). Sequence results were released on August 22, 2023 (GISAID accession EPI_ISL_18125249). Outgrowth to expand this virus was started on August 24, 2023, in the Vero-TMPRSS cell line, where two passages were performed (Methods). The sequence of the outgrown virus was deposited to GISAID (EPI_ISL_18226980) on September 6, 2023, with no in vitro sequence changes detected relative to the accepted Omicron BA.2.86 sequence, including no R682W in vitro spike mutation.

To test whether BA.2.86 can escape current population immunity, we compared neutralization of BA.2.86 to XBB.1.5 using sera from South African individuals who were infected during the period when XBB-descendent subvariants were dominant in South Africa (Fig. 1C, see Table S1 for summary participant information, Table S2 for participant details and Fig. S2 for the time periods when different variants/subvariants circulated in South Africa). We also used a second panel of sera which we collected from South African participants in September 2023 in a serosurvey of the current state of neutralizing antibody immunity (Fig. 1D, see Table S3 for summary participant information and Table S4 for participant details). Given the current complex state of immunity to SARS-CoV-2 in the South African population where multiple exposures are likely both because of infection^27^ and vaccination^17^, we did not stratify participants by age, gender, vaccine doses, or HIV status.

To determine live-virus neutralization capacity, we calculated the focus reduction neutralization test (FRNT_50_) value, which is the inverse of the plasma dilution required for 50% reduction in infection focus number. The panel of sera from XBB-family infected individuals, collected a median of 3 weeks post-infection (Table S1), showed a geometric mean titer (GMT) FRNT_50_ of 363 for XBB.1.5 and 220 for BA.2.86, a non-significant difference (Fig. 1C). In the serosurvey samples, which, unlike the samples used for the XBB panel, were not specifically taken close to peak immunity post-infection but rather reflect the current neutralizing immunity of the South African population at the time of writing, showed very low immunity for both subvariants, with GMT FRNT_50_ of 34 for XBB.1.5 and 42 for BA.2.86 (Fig. 1D). About a third of samples were below the level of quantification which was FRNT_50_ = 25. Excluding the samples below the level of quantification (LOQ), GMT FRNT_50_ was 96 for both XBB.1.5 and BA.2.86 (Fig. 1D, samples above LOQ in blue).

Next, we examined if this variant evolved escape to neutralizing immunity relative to earlier SARS-CoV-2 strains. We checked neutralization by vaccinated individual sera collected pre-Omicron (see Tables S5 and S6 for participant details) which we previously used to determine escape of the first Omicron subvariant, BA.1^3^. Here, we found over 100-fold escape of BA.2.86 relative to ancestral SARS-CoV-2, 5-fold greater than observed for BA.1 (Fig. 1E). We also tested for escape relative to Omicron BA.1 in people infected with BA.1 (see Table S7 and S8 for participant details). Here, again we found extensive escape which was 14-fold relative to BA.1. However, XBB.1.5 showed a 12-fold escape relative to BA.1, similar to the result we obtained with BA.2.86 (Fig. 1F).

**Fig. 2:**
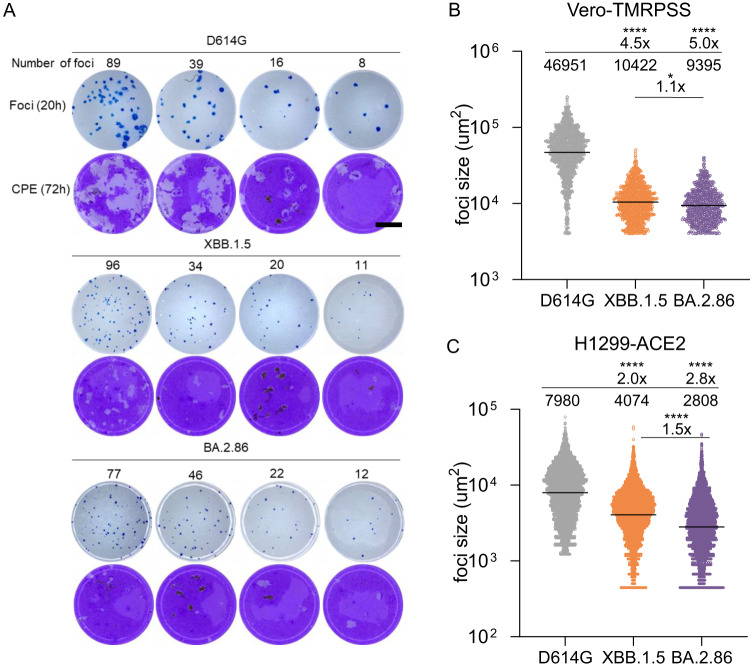
Omicron BA.2.86 infection in cell culture. **A** Foci formed by ancestral D614G, XBB.1.5, and BA.2.86 20 h post-infection on Vero-TMPRSS2 cells (rows 1, 3, 5) and cytopathic effect by the same viral inoculum at 72 h post-infection (rows 2, 4, 6). Rows 1 and 2 were infected with D614G, 3 and 4 with XBB.1.5, and 5 and 6 with BA.2.86. The number of foci per well is indicated above each well. **B** Quantitation of focus area for D614G, XBB.1.5, and BA.2.86 in Vero-TMPRSS2 cells. Bars are geometric means of focus area (*n* = 1389 for D614G, *n* = 1049 for XBB.1.5, *n* = 720 for BA.2.86) from three independent experiments. **C** Quantitation of focus area for D614G, XBB.1.5, and BA.2.86 in H1299-ACE2 cells. Bars are geometric means of focus area (*n* = 5016 for D614G, *n* = 5584 for XBB.1.5, *n* = 4921 for BA.2.86) from three independent experiments. Significant *p*-values were *p = 0.02, ****p < 10^−15^ by the two-sided Kruskal–Wallis test with Dunn multiple hypothesis correction.

We then investigated whether there were any differences in infection properties in Vero-TMPRSS2 cells, a cell line known to lack an interferon response, and the H1299 human lung epithelial cell line stably expressing the ACE2 receptor, which has been reported to have an interferon response^28–32^. We measured focus size (in the absence of neutralizing antibody), where one focus forms when the infection is spread from one infected cell to surrounding cells (Fig. S3). In Vero-TMPRSS2 cells, we found that both BA.2.86 and XBB.1.5 made infection foci which were 5-fold and 4.5-fold smaller in area relative to ancestral SARS-CoV-2 D614G at 20 h post-infection (Fig. 2A rows 1, 3, and 5, quantitation in Fig. 2B). The same viral inoculum led to considerably more cytopathic effect (CPE) by 72 h in ancestral SARS-CoV-2 infected cells relative to both BA.2.86 and XBB.1.5 (Fig. 2A rows 2, 4, and 6). In H1299-ACE2 cells, both BA.2.86 and XBB.1.5 still made significantly smaller foci relative to ancestral SARS-CoV-2, although the fold-difference was less (2-fold and 2.8-fold smaller for XBB.1.5 and BA.2.86, Fig. 2C). BA.2.86 foci were moderately but significantly smaller than XBB.1.5 foci.

We also measured replication in H1299-ACE2 cells as fold-change in viral genomes determined by quantitative polymerase chain reaction (qPCR) cycle threshold (Ct) values, where we found that fold-change by Ct by the kit used had a strong correlation to the number of infectious viruses (Fig. S4). We observed that ancestral SARS-CoV-2 initially replicated faster than either BA.2.86 and XBB.1.5 (1-day post-infection), consistent with the focus size data. However, both BA.2.86 and XBB.1.5 closed the gap and then overtook ancestral SARS-CoV-2 by day 4 post-infection (Fig. S5). We also estimated the infectiousness of the cell-free virus in terms of approximate viral genomes required for one infection focus and found that the cell-free BA.2.86 stock was less infectious in both cell lines relative to both XBB.1.5 and ancestral SARS-CoV-2 (higher genomes required per focus, Fig. S6). We note that the infectivity of a live viral stock may potentially be influenced by producer cell viability or other factors during virus production in vitro, which we did not control for.

## Discussion

Overall, our results indicate that, although the Omicron BA.2.86 subvariant has evolved extensive escape from neutralizing antibodies, it is recognized by convalescent plasma to a similar degree as the XBB.1.5 subvariant. This similarity in recognition might explain the comparatively slow spread of this variant (Fig. S2). These observations are broadly similar to other data showing moderate^33–35^ or no neutralization escape^36–40^ of BA.2.86 relative to recent circulating variants from neutralizing immunity of people recently exposed to SARS-CoV-2. The differences between our results and the studies showing moderate escape relative to XBB-derived subvariants may be related to the different populations from which study participants were drawn and their associated immune histories. As can be expected from the large number of mutations, BA.2.86 was reported to escape some of the monoclonal neutralizing antibodies which have activity against other recent variants, although it is more sensitive to other monoclonals^33,36,37^.

We did not observe substantial differences between BA.2.86 and XBB.1.5 in replication and spread between cells as detected by focus size, and no major differences were visible in the cytopathic effect. We did observe that more viral genomes were needed per cell for productive infection with BA.2.86 relative to the XBB.1.5 virus, consistent with other reports^33,34^.

Limitations to our conclusions include a lack of stratification into subgroups based on age, HIV status, and detailed immune history. Given the current heterogeneity in immune histories, such stratification leads to a comparison of very small groups. Our cohort is broadly representative of the South African population^41,42^ and our results should present the state of current neutralizing immunity in South Africa, which has distinct features relative to other locations such as less recent vaccination, no variant sequence vaccines, and higher HIV prevalence. A second limitation is that we used engineered cell lines that do not represent the full spectrum of virus-cell interactions: the Vero cell line which lacks an interferon response, and the H1299 cell line, having low levels of TMPRSS2 expression, has been shown to be predominantly infected by SARS-CoV-2 through the endocytic pathway^43^ and therefore would not detect differences in infection through the TMPRSS2-dependent plasma membrane pathway. Therefore, our data is not inconsistent with another report showing increased BA.2.86 infectivity relative to XBB.1.5 in Calu3 cells which do use the TMPRSS2 pathway^37^. Additional work in primary cells and animal models will give greater clarity on cell tropism, pathogenicity, and replication of BA.2.86 relative to other circulating strains. An additional limitation is that we did not compare BA.2.86 to a more recent subvariant such as the Omicron XBB.1.9 derived EG.5.1.

Our phylogenetic analysis suggests that BA.2.86 descends from viruses that circulated in early 2022 without any observed intermediates and only started to spread recently. During the period of unobserved evolution (~16 months from early 2022 to May 2023), the viral genome accumulated at least 47 nucleotide changes and several deletions, and one insertion. This rate of evolution (~35 changes per year) is about two-fold larger than that observed on short time scales in circulating variants^26^, but consistent with the rate along branches leading to other major variants^26^. There may be several explanations for the long period of evolution in the absence of population spread, including evolution in long-term SARS-CoV-2 infection during immunosuppression due to factors such as advanced HIV disease^44–46^ as well as infection in an animal reservoir^46,47^.

Our data indicates that, phenotypically, BA.2.86 is not very different from SARS-CoV-2 strains already in circulation. However, despite the lack of substantial differences compared to Omicron subvariant XBB.1.5, we do see very low levels of current neutralizing antibody immunity to BA.2.86 in the South African population. This opens the possibility for BA.2.86 to evolve further immune escape and replicative capacity.

## Methods

### Informed consent and ethical statement

Sex and/or gender were not considered in the study design as enrollment was of all SARS-CoV-2 infected participants. Blood samples and nasopharyngeal swabs for ancestral D614G SARS-CoV-2 isolation, as well as all blood samples used in the neutralization experiments, were obtained after written informed consent from adults with PCR-confirmed SARS-CoV-2 infection who were enrolled in a prospective cohort study at the Africa Health Research Institute approved by the Biomedical Research Ethics Committee at the University of KwaZulu–Natal (reference BREC/00001275/2020). Blood samples were collected from 21 participants with Omicron XBB-derived infection (13 female, 8 male), age range 28–83. For samples used in the serosurvey analysis, blood samples were collected from 40 participants (33 female, 7 male) age range 18–61. For pre-Omicron vaccinated participants, blood samples were collected from 19 participants (12 females, 7 males) age range of 22–75. For the BA.1 infected participants, blood samples were collected from 19 participants (14 female, 5 male) with age range 26-81. Participants received compensation for each study visit as approved by the Biomedical Research Ethics Committee at the University of KwaZulu–Natal. The Omicron/BA.1 and BA.2.86 were isolated from a residual swab sample with SARS-CoV-2 isolation from the sample approved by the University of the Witwatersrand Human Research Ethics Committee (HREC) (ref. M210752). The sample to isolate XBB.1.5 was collected after written informed consent as part of the COVID-19 transmission and natural history in KwaZulu–Natal, South Africa: Epidemiological Investigation to Guide Prevention and Clinical Care in the Center for the AIDS Program of Research in South Africa (CAPRISA) study and approved by the Biomedical Research Ethics Committee at the University of KwaZulu–Natal (reference BREC/00001195/2020, BREC/00003106/2021).

### Whole-genome sequencing and genome assembly

For the BA.2.86 swab sample, RNA was extracted on an automated Chemagic 360 instrument, using the CMG-1049 kit (Perkin Elmer, Hamburg, Germany). Libraries for whole-genome sequencing were prepared using the Illumina COVIDseq Assay (Illumina Inc, San Diego, CA) and version 4 SARS-CoV-2 primer pools. Pooled PCR products were fragmented and tagged to adapter sequences. The adapter-tagged amplicons were purified and indexed using sets 1–4 of PCR indexes (Illumina). Libraries were quantified using a Qubit 4.0 fluorometer (ThermoFisher Scientific, Oregon, USA) using the Qubit dsDNA High Sensitivity assay according to the manufacturer’s instructions. Fragment sizes were analyzed using the TapeStation 4200 system (Agilent Technologies, Santa Clara, CA). Libraries were pooled and normalized to 4 nM sample library with a 2% PhiX spike-in. Libraries were loaded onto a 300-cycle NextSeq P2 Reagent Kit v2 and run on the Illumina NextSeq 1000/2000 instrument (Illumina). Sequencing data was analyzed using Exatype v4.1.5 (Hyrax Biosciences, Cape Town, South Africa) with default parameters (10% minimum prevalence to report variants, 80% minimum prevalence to include a variant in consensus sequence). Nextclade (v2.14.1) and Pangolin (v4.3, Pangolin-data v1.21) were used for clade and lineage assignments. Additionally, Nextclade was used for the visualization of the sequences and the identification of frameshifts. Unknown frameshifts were manually corrected using Aliview (v1.24). Outbreak.info was used to determine the prevalence of mutations.

For the BA.2.86 outgrowth sample, Oxford Nanopore sequencing was performed. RNA was manually extracted from either 200 µL input volume using either the MagMAX™ Viral/Pathogen II Nucleic Acid Isolation Kit (Thermo Scientific, A42352) or from 140 µL using the QIAamp Viral RNA Kit (Qiagen, 52906) as per the manufacturer’s protocols. All RNA extractions were measured using Qubit fluorimeter kits (Thermo Scientific, Q32852). The cDNA synthesis was performed using LunaScript RT mastermix (New England BioLabs) followed by whole-genome multiplex PCR using the Midnight Primer pools v3 (EXP-MRT001, Oxford Nanopore) that produce 1200-base-pair amplicons. The amplified products for each pool were combined and used for library preparation procedures using the Oxford Nanopore Rapid Barcoding kit (SQK-RBK110.96, Oxford Nanopore). The barcoded samples were pooled and cleaned up using magnetic beads and loaded on an R9.4.1 flow cell for 8-h sequencing on a MinION device. The raw data was processed using Guppy basecaller and Guppy barcoder (Oxford Nanopore) for basecalling and demultiplexing. The final consensus sequences were obtained using the Genome Detective v2.64. The lineage assignment was determined using Nextclade.

### Phylogenetic analysis

We assembled a set of 280 BA.2 (Nextstrain clade 21 L) sequences collected between November 2021 and June 2022 from data deposited on GISAID^48^. BA.2.86 sequences were downloaded on September 7 2023 directly from GISAID. We excluded sequences with reversion mutations relative to BA.2, sequences flagged as poor quality by Nextclade^49^, or sequences with less than 90% coverage of the reference. Sequences were pairwise aligned against Wuhan-Hu-1 using Nextclade. Terminals and gaps were masked as well as all suspected artefactual reversions to reference in BA.2.86 sequences. A tree was built using IQ-tree 2^50^ and post processed using a custom script to correct for incomplete merging of branches in large polytomies.

A time tree was inferred using TreeTime^51^ using a clock rate of 0.0005 per site and year^26^. The rate of the long branch between BA.2 and BA.2.86 was set to be 2 times the rate of the rest of the tree in line with a previous observation that evolution is 2-fold accelerated along many long branches leading to distinct clades^26^. This acceleration is consistent with the dramatic enrichment of amino acid substitutions in the spike protein along the long branch leading to BA.2.86.

### Cells

The VeroE6 cells expressing TMPRSS2 and ACE2 (VeroE6-TMPRSS2), originally BEI Resources, NR-54970 were used for virus expansion and all live-virus assays excluding replication. The Vero-TMPRSS2 cell line was propagated in growth medium consisting of Dulbecco’s Modified Eagle Medium (DMEM, Gibco 41965-039) with 10% fetal bovine serum (Hyclone, SV30160.03) containing 10 mM of hydroxyethylpiperazine ethanesulfonic acid (HEPES, Lonza, 17-737E), 1 mM sodium pyruvate (Gibco, 11360-039), 2mM L-glutamine (Lonza BE17-605E) and 0.1 mM nonessential amino acids (Lonza 13-114E). The H1299-E3 (H1299-ACE2, clone E3) cell line used in the replication assay was derived from H1299 (CRL-5803) and propagated in a growth medium consisting of complete Roswell Park Memorial Institute (RPMI, Gibco, 21875-034) 1640 with 10% fetal bovine serum containing 10 mM of HEPES, 1 mM sodium pyruvate, 2mM l-glutamine and 0.1 mM nonessential amino acids.

### Virus expansion

All work with live virus was performed in Biosafety Level 3 containment using protocols for SARS-CoV-2 approved by the Africa Health Research Institute Biosafety Committee. VeroE6-TMPRSS2 cells were seeded at 4.5 × 10^5^ cells in a 6-well plate well and incubated for 18–20 h pre-infection. After one Dulbecco’s phosphate-buffered saline (DPBS) wash, the sub-confluent cell monolayer was inoculated with 500 μL with universal transport medium which contained the swab, diluted 1:2 with growth medium filtered through a 0.45 μm and 0.22 μm filters. Cells were incubated for 2 h. Wells were then filled with 3 mL complete growth medium. After 3 days of infection (completion of passage 1 (P1)), the supernatant was collected, cells were trypsinized, centrifuged at 300 × *g* for 3 min, and resuspended in 3 mL growth medium. All infected cells and supernatant were added to VeroE6-TMPRSS2 cells that had been seeded at 1.5 × 10^5^ cells per mL, 20 mL total, 18–20 h earlier in a T75 flask for cell-to-cell infection. The coculture was incubated for 1 h and the flask was filled with 20 mL of complete growth medium and incubated for 3 days. The viral supernatant from this culture (passage 2 (P2) stock) was used for experiments.

### Live-virus focus-forming assay and neutralization assay

For all neutralization assays, viral input was 100 focus-forming units per well of a 96-well plate. VeroE6-TMPRSS2 cells were plated in a 96-well plate (Corning) at 30,000 cells per well 1-day pre-infection. Plasma was separated from EDTA-anticoagulated blood by centrifugation at 500 × *g* for 10 min and stored at −80 °C. Aliquots of plasma samples were heat-inactivated at 56 °C for 30 min and clarified by centrifugation at 10,000 × *g* for 5 min. Virus stocks were used at approximately 50–100 focus-forming units per microwell and added to diluted plasma in neutralization assays. Antibody–virus mixtures were incubated for 1 h at 37 °C, 5% CO_2_. Cells were infected with 100 μL of the virus–antibody mixtures for 1 h, then 100 μL of a 1X RPMI 1640 (Sigma-Aldrich, R6504), 1.5% carboxymethylcellulose (Sigma-Aldrich, C4888) overlay was added without removing the inoculum. Cells were fixed 20 h post-infection using 4% PFA (Sigma-Aldrich, P6148) for 20 min. Foci were stained with a rabbit anti-spike monoclonal antibody (BS-R2B12, GenScript A02058) at 0.5 μg/mL in a permeabilization buffer containing 0.1% saponin (Sigma-Aldrich, S7900), 0.1% BSA (Biowest, P6154) and 0.05% Tween-20 (Sigma-Aldrich, P9416) in PBS for 2 h at room temperature with shaking, then washed with wash buffer containing 0.05% Tween-20 in PBS. A secondary goat anti-rabbit HRP conjugated antibody (Abcam ab205718) was added at 1 μg/mL and incubated for 2 h at room temperature with shaking. TrueBlue peroxidase substrate (SeraCare 5510-0030) was then added at 50 μL per well and incubated for 20 min at room temperature. Plates were imaged in an ImmunoSpot Ultra-V S6-02-6140 Analyzer ELISPOT instrument with BioSpot Professional built-in image analysis (C.T.L) which was also used to quantify areas of individual foci.

### Statistics and fitting

All statistics were performed in GraphPad Prism version 9.4.1. All fitting to determine FRNT_50_ and linear regression was performed using custom code in MATLAB v.2019b (FRNT_50_) or the fitlm function for linear regression, which was also used to determine goodness-of-fit (*R*^2^) as well as *p*-value by *F*-test of the linear model.

Neutralization data were fit to:

1 $${{{{{\mathrm{TX}}}}}}=1/1+(D/{{{{{\mathrm{I}}}}}}{{{{{\mathrm{{D}}}}}}}_{50})$$

Here, Tx is the number of foci at plasma dilution *D* normalized to the number of foci in the absence of plasma on the same plate. ID_50_ is the plasma dilution giving 50% neutralization. FRNT_50_ = 1/ID_50_. Values of FRNT_50_ < 1 are set to 1 (undiluted), the lowest measurable value. We note that the most concentrated plasma dilution was 1:25 and therefore FRNT_50_ < 25 was extrapolated.

### Plaque assay

VeroE6-TMPRSS2 cells were plated in a 96-well plate (Corning) at 30,000 cells per well 1-day pre-infection. Virus stocks (used at the focus-forming units per microwell shown in Fig. 2) were added to cells, and incubated for 1 h at 37 °C, 5% CO2. Following incubation, 100 μL of a 1X RPMI 1640 (Sigma-Aldrich, R6504), 1.5% carboxymethylcellulose (Sigma-Aldrich, C4888) overlay was added without removing the inoculum. Cells were fixed 72 h post-infection using 4% PFA (Sigma-Aldrich, P6148) for 20 min. The fixed cells were washed with distilled water and stained with 30 μL/well of a 0.5% crystal violet solution (Sigma-Aldrich, 61135).

### Replication assay

H1299-E3 cells were seeded at 1 × 10^6^ cells in a 5 mL growth medium in a Corning T25 flask 18–20 h pre-infection. Cells were infected with 5 focus-forming units of either ancestral B.1, Omicron XBB.1.5, or Omicron BA.2.86. 300 µL of supernatant was collected at the input (day 0) and on days 1–4 post-infection.

### Cycle threshold values for SARS-CoV-2 RNA copies

Samples were diluted 1:3 with PBS and sent to an accredited diagnostic laboratory (Molecular Diagnostic Services, Durban, South Africa) to determine SARS-CoV-2 cycle threshold (Ct) values. At Molecular Diagnostic Services, samples were extracted using a guanidine isothiocyanate/magnetic bead-based method with the NucliSense (Biomerieux) extractor of the KingFisher Flex 96 (Thermo Fisher). Reverse transcription-quantitative polymerase chain reaction (RT-qPCR) was performed using the Seegene Allplex 2019 nCoV assay with the Bio-Rad CFX96 real-time PCR instrument as per the kit instructions. RNase P is used as the internal housekeeping gene to monitor extraction and assay efficiency. The kit targets the E, N, and R genes of SARS-CoV-2. Run calls and interpretation were performed by the Seegene Viewer software. Fold-change was calculated as FC = 2^((mean(Ct input) – Ct sample)^ in the replication experiment and FC = 2^(Ct most dilute sample – Ct sample)^.

### Cycle threshold linearity and infectivity assay

To determine whether Ct values were a good correlate for infectious viruses and to determine infectivity (number of viral genomes per focus-forming unit), 2-fold serial dilutions of a viral stock starting at approximately 100 focus-forming units were sent to determine Ct as above and in parallel plated to determine focus-forming units. For infectivity, fold-change was determined from the Ct as above, and linear regression was performed using MATLAB v.2019b against the focus-forming units obtained for the same dilution. To determine the number of viral genomes required per focus-forming unit, Ct values were converted to viral genomes using the approximation from ref. ^52^. Regression was then performed using MATLAB v.2019b against the focus-forming units obtained for the same dilution.

### Reporting summary

Further information on research design is available in the Nature Portfolio Reporting Summary linked to this article.

### Supplementary information

Supplementary Information

Peer Review File

Reporting Summary

## Data Availability

Viral isolates are available upon reasonable request. Source data are provided in this paper. Sequences of isolated SARS-CoV-2 used in this study have been deposited in GISAID and GenBank with accession numbers as follows: Virus GISAID GenbankID Hyperlink D614G (B.1 lineage) EPI_ISL_602626.1 OP090658. XBB.1.5 EPI_ISL_17506815 OR782922. BA.2.86 EPI_ISL_18226980 OR775659. BA.1 EPI_ISL_7886688 OP090659. All genome sequences and associated metadata in this dataset are published in GISAID’s EpiCoV database (GISAID Identifier: EPI_SET_231003fr). To view the contributors of each individual sequence with details such as accession number, Virus name, Collection, Originating Lab and Submitting Lab, and the list of Authors, visit 10.55876/gis8.231003fr. EPI_SET_231003fr is composed of 625 individual genome sequences. The collection dates range from 2021-12-14 to 2023-09-27; Data were collected in 48 countries and territories; All sequences in this dataset are compared relative to hCoV-19/Wuhan/WIV04/2019 (WIV04), the official reference sequence employed by GISAID (EPI_ISL_402124). For more information https://gisaid.org/WIV04.

## Code availability

Image analysis and curve fitting scripts in MATLAB v.2019b are available on GitHub (https://github.com/sigallab/NatureMarch2021). The phylogenetic workflow is available at github.com/neherlab/BA286. The repository contains a specific list of sites (config/mask.tsv) that are masked in individual sequences. An interactive version of the phylogenetic tree is available at https://nextstrain.org/groups/neherlab/ncov/BA.2.86. The code is also available at *Zenodo*, 10.5281/zenodo.10082570.
