# Supplementary material for: Evolution and neutralization escape of the SARS-CoV-2 BA.2.86 subvariant
Source: Nat Commun. 2023 Dec 6;14:8078. doi: 10.1038/s41467-023-43703-3 (PMC10700484; doi:10.1038/s41467-023-43703-3)
Supplement: Supplementary file 1 — Supplementary Information [file 41467_2023_43703_MOESM1_ESM.pdf]

Supplementary Material

Evolution and neutralization escape of the SARS-CoV-2 BA.2.86 subvariant

Khan et al.

Contents

Table S1: Summary Omicron XBB derived variant infected participants 2
Table S2: Omicron XBB derived variant infected participants 2
Table S3: Summary for September 2023 serosurvey 3
Table S4: Serosurvey participants enrolled in September 2023 3
Table S5: Summary for pre-Omicron Pfizer BNT162b2 vaccinated participants 4
Table S6: Pre-Omicron participants vaccinated with Pfizer BNT162b2 4
Table S7: Summary for Omicron BA.1 infected participants 5
Table S8: Omicron BA.1 infected participants 5
Figure S1: Phylogenetic structure of BA.2.86 spread 6
Figure S2: Timing of the different variant infection waves in South Africa 7
Figure S3: Magnified view of infection foci 8
Figure S4: Relationship of focus forming units to fold-change as determined by qPCR in a dilution series 9
Figure S5: BA.2.86 replication in H1299-ACE2 cells 10
Figure S6: Estimation of infectivity 11

Table S1: Summary Omicron XBB derived variant infected participants

	n=21
Age (median, IQR)	45 (31-63)
Female	13 (62%)
Days post-infection to sample (median, IQR)	21 (14-29)
Fraction vaccinated	13 (62%)
PLWH	7 (33%)

Table S2: Omicron XBB derived variant infected participants

#	Sex	Age range	Sample collection date	Infection date	Infect. to sample (days)	Vacc. Status	Vacc. doses	Last vaccine	HIV status	XBB.1.5 FRNT50	BA.2.86 FRNT50
1	F	30-39	May 23	Apr 23	14	mRNA	2	Jul 21	-	629	349
2	F	50-59	Jun 23	May 23	29	mRNA	3	Sep 21	-	924	629
3	F	20-29	Jun 23	Jun 23	15	Ad26	2	May 21	-	549	323
4	M	60-69	Sep 23	Aug 23	23	Ad26/Moderna	2	Oct 22	-	80	58
5	F	20-29	May 23	Apr 23	21	Ad26	2	Dec 21	-	1127	764
6	F	60-69	May 23	May 23	13	mRNA	3	Mar 22	+	505	404
7	F	70-79	May 23	May 23	7	mRNA	2	Nov 21	-	3262	2175
8	M	40-49	Jun 23	May 23	24	Ad26/mRNA	2	Nov 22	-	948	354
9	F	80-89	Jun 23	May 23	29	mRNA	2	Jun 21	-	458	128
10	M	70-79	Oct 22	Oct 22	14	mRNA	2	Aug 21	-	2522	1273
11	F	30-39	Feb 23	Jan 23	35	mRNA	2	Oct 21	+	11	8
12	M	30-39	Mar 23	Feb 23	30	mRNA	2	Jun 21	-	234	50
13	F	40-49	May 23	Apr 23	15	Ad26	1	May 21	+	113	110
14	M	50-59	May 23	Apr 23	12	Unvacc.	–	–	-	10239	1736
15	F	40-49	Feb 23	Jan 23	33	Unvacc.	–	–	-	276	134
16	F	30-39	Mar 23	Mar 23	17	Unvacc.	–	–	+[#]	2003	1770
17	M	60-69	Apr 23	Apr 23	28	Unvacc.	–	–	-	956	39
18	M	20-29	Jun 23	May 23	22	Unvacc.	–	–	+[&]	58	171
19	F	30-39	May 23	Mar 23	37	Unvacc.	–	–	+	9	27
20	F	40-49	Jun 23	May 23	21	Unvacc.	–	–	+	25	50
21	M	30-39	Jun 23	Jun 23	14	Unvacc.	–	–	-	502	600

Infection date is by date of first available positive qPCR test. All participants living with HIV were HIV suppressed (HIV viral load <200 copies/mL) except for [#], where HIV viral load = 48685 and [&] where HIV viral load = 89040. mRNA: Pfizer-BioNTech BNT162b2 mRNA vaccine. Ad26: Janssen Ad26.COV2.S vectored vaccine. All vaccines based on ancestral spike sequence.

Table S3: Summary for September 2023 serosurvey

	n=40
Age (median, IQR)	29 (25-36)
Female	33 (83%)
Fraction vaccinated	31 (78%)

Vaccination status unavailable for 2 participants, HIV status was not tested.

Table S4: Serosurvey participants enrolled in September 2023

#	Sex	Age Range	Vacc. Status	Vacc. doses	Last vaccine	XBB.1.5 FRNT50	BA.2.86 FRNT50
**1**	F	20-29	Unvacc.	–	–	332	125
**2**	M	30-39	Unvacc.	–	–	14	9
**3**	F	20-29	Ad26	1	Aug 21	26	14
**4**	F	20-29	Ad26	1	Mar 21	54	6
**5**	F	20-29	Ad26	1	Mar 21	42	163
**6**	F	20-29	Ad26	1	Jun 22	246	565
**7**	F	20-29	Ad26	1	Jun 22	135	320
**8**	F	20-29	Ad26	1	Jun 21	8	10
**9**	F	20-29	Unvacc.	–	–	184	220
**10**	F	20-29	N/A	N/A	N/A	141	268
**11**	F	20-29	mRNA	2	Feb 22	24	24
**12**	F	20-29	Unvacc.	–	–	223	54
**13**	F	60-69	Ad26	1	Mar 21	168	151
**14**	F	20-29	Unvacc.	–	–	68	105
**15**	F	20-29	Unvacc.	–	–	17	9
**16**	F	20-29	Ad26	1	Feb 22	90	62
**17**	M	50-59	Ad26	1	Mar 21	44	52
**18**	F	10-19	Ad26	1	Mar 22	22	46
**19**	F	10-19	Ad26	2	Jan 22	1	4
**20**	M	30-39	Unvacc.	–	–	154	73
**21**	F	30-39	Ad26	2	Mar 23	84	65
**22**	F	20-29	Ad26	1	N/A	97	107
**23**	F	30-39	mRNA	2	N/A	5	3
**24**	F	40-49	Ad26	1	N/A	193	164
**25**	F	40-49	Ad26	2	N/A	206	156
**26**	F	20-29	Ad26	2	N/A	74	90
**27**	F	40-49	mRNA	2	N/A	17	72
**28**	F	30-39	mRNA	3	N/A	38	51
**29**	F	20-29	Ad26	1	May 21	9	7
**30**	F	30-39	mRNA	3	N/A	55	12
**31**	M	20-29	Ad26	1	Aug 22	5	9
**32**	M	20-29	Ad26	1	N/A	2	12
**33**	F	30-39	mRNA	2	Aug 21	236	271
**34**	F	50-59	Ad26	1	N/A	27	25
**35**	M	40-49	Ad26	1	N/A	5	10
**36**	M	30-39	mRNA	2	N/A	1	9
**37**	F	30-39	mRNA	2	N/A	38	75
**38**	F	50-59	N/A	N/A	N/A	122	50
**39**	F	20-29	mRNA	3	N/A	1	30
**40**	F	30-39	mRNA	2	N/A	17	35

mRNA: Pfizer-BioNTech BNT162b2 mRNA vaccine. Ad26: Janssen Ad26.COV2.S vectored vaccine. All vaccines based on ancestral spike sequence. N/A – not available.

Table S5: Summary for pre-Omicron Pfizer BNT162b2 vaccinated participants

	n=19
Age (median, IQR)	52 (41-67)
Female	12 (63%)
Days post-vaccination to sample (median, IQR)	28 (14-33)
PLWH	8 (42%)

Table S6: Pre-Omicron participants vaccinated with Pfizer BNT162b2

#	Sex	Age range	Sample collected date	Vacc. doses	Last vaccine	Vacc. to sample (days)	HIV status	D614G FRNT50	BA.1 FRNT50	BA.2.86 FRNT50
**1**	F	40-49	Sep 21	2	Aug 21	32	+	942	11	10
**2**	F	40-49	Oct 21	2	Sep 21	34	+	16920	984	76
**3**	F	60-69	Oct 21	2	Aug 21	63	-	11789	621	68
**4**	M	40-49	Oct 21	2	Oct 21	18	+	9629	750	37
**5**	M	70-79	Aug 21	2	Jun 21	38	-	5025	138	36
**6**	F	60-69	Jul 21	2	Jul 21	11	-	301	16	10
**7**	M	70-79	Jul 21	2	Jul 21	11	-	720	45	9
**8**	M	30-39	Aug 21	2	Jul 21	15	+	894	44	10
**9**	F	70-79	Jul 21	2	Jul 21	10	-	301	24	17
**10**	F	30-39	Jul 21	2	Jul 21	11	+	2002	76	16
**11**	F	20-29	Nov 21	2	Oct 21	31	-	3625	235	13
**12**	M	20-29	Nov 21	2	Oct 21	30	-	2963	104	28
**13**	F	60-69	Aug 21	2	Jul 21	28	-	59259	1143	74
**14**	M	60-69	Aug 21	2	Jul 21	26	-	71412	2780	117
**15**	F	40-49	Nov 21	2	Oct 21	33	+	15276	1105	328
**16**	M	50-59	Oct 21	2	Sep 21	30	-	21376	759	73
**17**	F	50-59	Nov 21	2	Oct 21	13	-	2915	385	91
**18**	F	60-69	Nov 21	2	Nov 21	14	+	12700	174	118
**19**	F	30-39	Aug 21	2	Jul 21	34	+	259	5	5

All participants living with HIV were HIV suppressed (HIV viral load <200 copies/mL).

Table S7: Summary for Omicron BA.1 infected participants

	n=19
Age (median, IQR)	51 (35-57)
Female	14 (74%)
Days post-infection to sample (median, IQR)	23 (21-24)
Fraction vaccinated	11 (58%)
PLWH	8 (42%)

Table S8: Omicron BA.1 infected participants

#	Sex	Age range	Sample collection date	Infection date	Infect. to sample (days)	Vacc. Status	Vacc. doses	Last vaccine	HIV status	BA.1 FRNT50	XBB.1.5 FRNT50	BA.2.86 FRNT50
1	M	50-59	Jan 22	Dec 21	17	mRNA	2	Aug 21	+	1336	256	186
2	M	30-39	Dec 21	Dec 21	23	Ad26	1	Mar 21	-	732	43	41
3	M	30-39	Dec 21	Nov 21	21	Ad26	2	May 21	-	1283	17	12
4	F	50-59	Dec 21	Dec 21	23	mRNA	2	Jul 21	+	756	63	24
5	F	30-39	Jan 22	Dec 21	22	Ad26	2	Apr 21	+	899	177	169
6	F	60-69	Jan 22	Dec 21	24	mRNA	2	Jul 21	-	1222	84	53
7	F	50-59	Jan 22	Dec 21	23	Ad26	1	Jul 21	+	522	83	49
8	M	30-39	Dec 21	Dec 21	27	mRNA	2	Jul 21	-	486	17	15
9	F	30-39	Jan 22	Dec 21	21	Ad26	1	Aug 21	-	619	35	12
10	F	80-89	Jan 22	Jan 22	22	mRNA	2	Jul 21	-	1211	469	170
11	M	60-69	Jan 22	Dec 21	24	mRNA	2	Jul 21	-	265	84	70
12	F	30-39	Jan 22	Dec 21	23	Unvacc.	N/A		+	7963	187	207
13	F	20-29	Dec 21	Dec 21	21	Unvacc.	N/A		-	540	36	69
14	F	20-29	Dec 21	Dec 21	21	Unvacc.	N/A		-	345	46	55
15	F	50-59	Jan 22	Dec 21	31	Unvacc.	N/A		+	290	77	64
16	F	60-69	Jan 22	Dec 21	17	Unvacc.	N/A		-	826	34	66
17	F	50-59	Jan 22	Dec 21	27	Unvacc.	N/A		+	317	26	40
18	F	50-59	Jan 22	Dec 21	21	Unvacc.	N/A		-	1047	50	30
19	F	50-59	Jan 22	Dec 21	31	Unvacc.	N/A		+	2001	158	134

Infection date is by date of first available positive qPCR test. All participants living with HIV were HIV suppressed (HIV viral load <200 copies/mL). mRNA: Pfizer-BioNTech BNT162b2 mRNA vaccine. Ad26: Janssen Ad26.COV2.S vectored vaccine. All vaccines based on ancestral spike sequence.

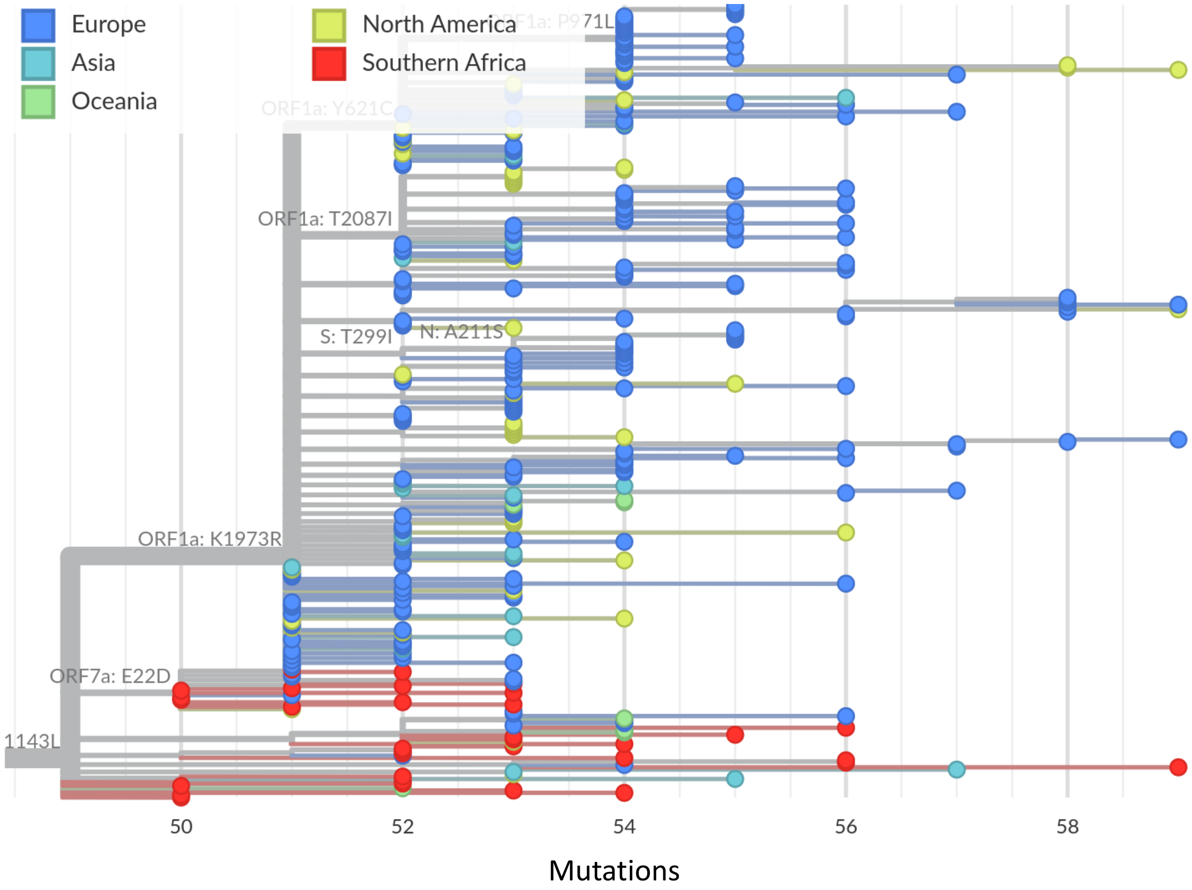

Figure S1: Phylogenetic structure of BA.2.86 spread. BA.2.86 differs by 49 single nucleotide mutations from BA.2. Within BA.2.86, most samples from the Northern Hemisphere fall into the large polytomy at the top. The majority of sequences differ by 3-7 mutations from the inferred MRCA of BA.2.86 with some sequences differing only by one mutation, others by up to 10 mutations.

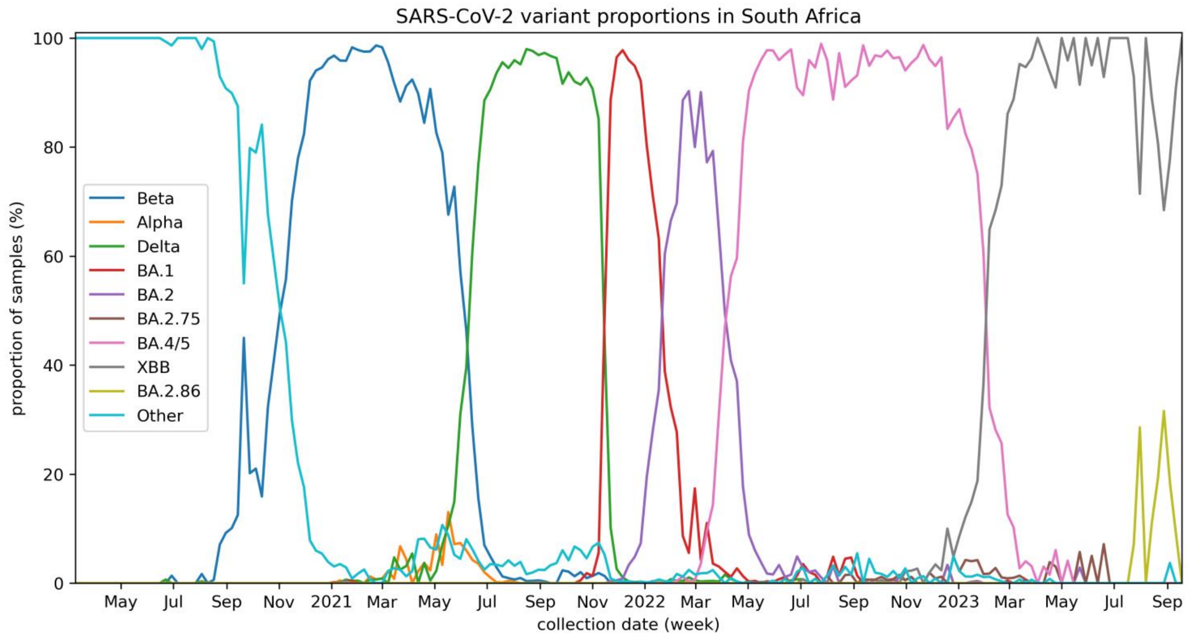

Figure S2: Timing of the different variant infection waves in South Africa. Plot of relative variant frequencies in South African consensus sequences binned by calendar week. Sequences deposited in GISAID were assigned to broad variant categories using a combination of Nextstrain clades and Pango lineages as called by Nextclade. The code used to produce the figure is available at https://github.com/neherlab/BA286/tree/master/variant-prevalence.

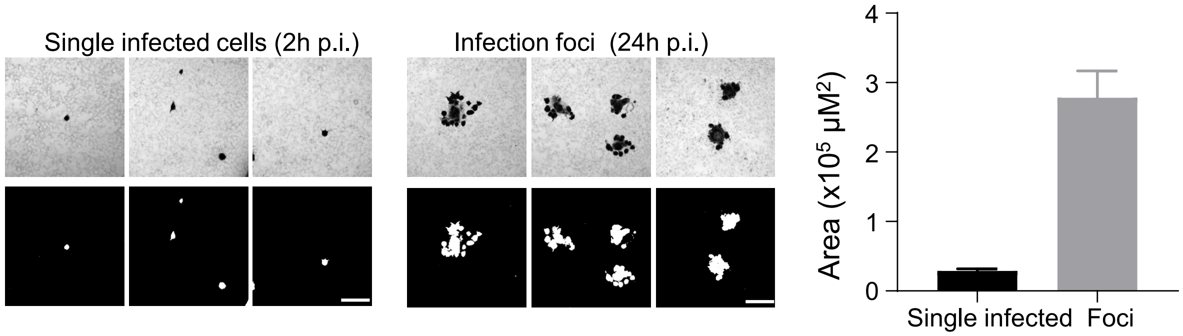

Figure S3: Magnified view of infection foci. Shown are three representative fields of view for infected H1299-ACE2 cells 2 hours post-addition of single infected cells to an uninfected cell layer (left set of images) and infection foci at 24 hours post-infection (right set of images). Bottom images are the masked images showing the antibody labelled infected cell or focus as white. Scale bar is 200 µm. Bar plot shows single infected cell and focus area (mean and standard deviation). Results are from 10 fields of view for single infected cells and 5 fields of view for foci.

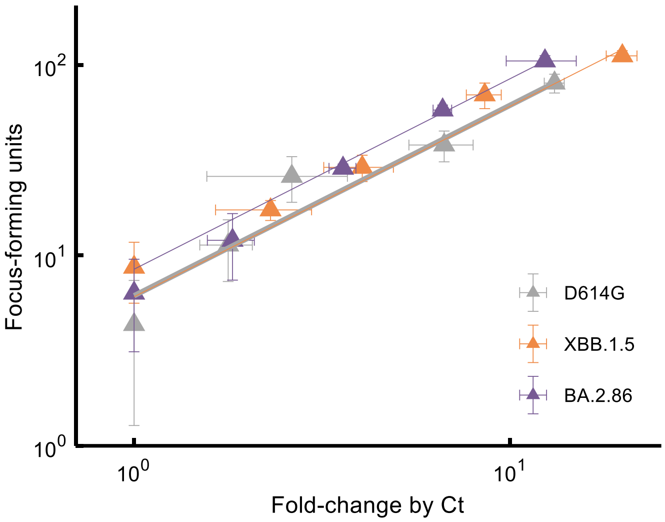

Figure S4: Relationship of focus forming units to fold-change as determined by qPCR in a dilution series. Ancestral SARS-CoV-2, XBB.1.5 and BA.2.86 were 2-fold serially diluted starting from a viral concentration which results in approximately 100 foci per well of 96-well plate and used to infect H1299-ACE2 cells. The serial dilution was assayed both for Ct and for focus forming units. Shown are mean and standard deviation from 3 independent experiments, with fold-change calculated as $2^{(CtMostDiluteSample-CtSample)}$. Solid lines correspond to linear regression fits to the data. Goodness-of-fit values for the linear model and p-values were $R^2 = 0.972$, $p = 6 \times 10^{-5}$ for ancestral SARS-CoV-2, $R^2 = 0.953$, $p = 2 \times 10^{-4}$ for XBB.1.5, and $R^2 = 0.996$, $p = 9 \times 10^{-7}$ for BA.2.86.

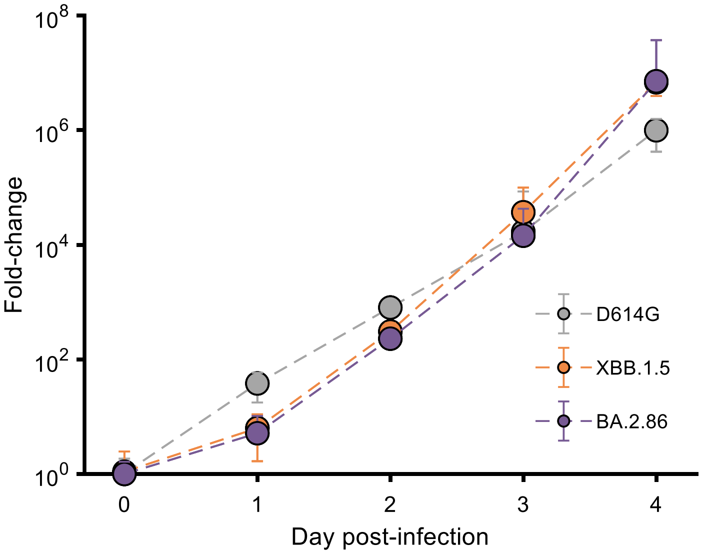

Figure S5: BA.2.86 replication in H1299-ACE2 cells. Fold-change in SARS-CoV-2 viral copies was determined using qPCR cycle threshold over 4 days of infection in H1299-ACE2 cells infected by 2 focus forming units per 10^6 cells (5 focus forming units total) using either ancestral SARS-CoV-2, XBB.1.5, or BA.2.86. Shown are median and interquartile ranges for 6 measurements from 3 independent experiments, with fold-change calculated as $2^{\overline{CtInput}-CtSample}$, with input being the most dilute sample.

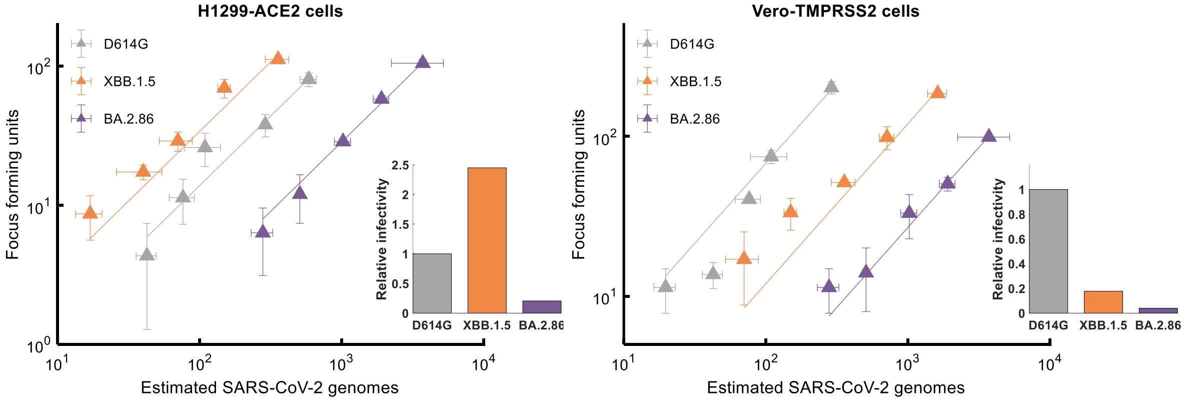

Figure S6: Estimation of infectivity. Ancestral SARS-CoV-2, XBB.1.5 and BA.2.86 were 2-fold serially diluted starting from a viral concentration which results in approximately 100 foci per well of 96-well plate and used to infect H1299-ACE2 cells (left) and Vero-TMPRSS2 cells (right). The serial dilution was assayed both for Ct and for focus forming units. Genome copies per μL were estimated from qPCR Ct values. Unweighted linear regression with y-intercept=0 was performed and is shown as solid lines. Goodness-of-fit values for the linear model and p-values were $R^2 = 0.972$, $p = 6 \times 10^{-5}$ for ancestral SARS-CoV-2, $R^2 = 0.953$, $p = 2 \times 10^{-4}$ for XBB.1.5, and $R^2 = 0.996$, $p = 9 \times 10^{-7}$ for BA.2.86 in H1299-ACE2 cells, and $R^2 = 0.982$, $p = 2 \times 10^{-5}$ for ancestral SARS-CoV-2, $R^2 = 0.973$, $p = 7 \times 10^{-5}$ for XBB.1.5, and $R^2 = 0.992$, $p = 5 \times 10^{-6}$ for BA.2.86 in Vero-TMPRSS2 cells. Slopes correspond to the number of foci produced per genome copy per μL for each viral strain. Relative infectivity was calculated by normalizing slope for each viral strain to that of ancestral SARS-CoV-2 and is shown as insets.
