# Supplementary material for: Evolution and neutralization escape of the SARS-CoV-2 BA.2.86 subvariant
Source: Nat Commun. 2023 Dec 6;14:8078. doi: 10.1038/s41467-023-43703-3 (PMC10700484; doi:10.1038/s41467-023-43703-3)
Supplement: Supplementary file 3 — Reporting Summary [file 41467_2023_43703_MOESM3_ESM.pdf]

nature portfolio

Corresponding author(s): Alex Sigal

Last updated by author(s): Nov 9, 2023

Reporting Summary

Nature Portfolio wishes to improve the reproducibility of the work that we publish. This form provides structure for consistency and transparency in reporting. For further information on Nature Portfolio policies, see our Editorial Policies and the Editorial Policy Checklist.

Statistics

For all statistical analyses, confirm that the following items are present in the figure legend, table legend, main text, or Methods section.

n/a	Confirmed	
☐	☒	The exact sample size (n) for each experimental group/condition, given as a discrete number and unit of measurement
☐	☒	A statement on whether measurements were taken from distinct samples or whether the same sample was measured repeatedly
☐	☒	The statistical test(s) used AND whether they are one- or two-sided<br>*Only common tests should be described solely by name; describe more complex techniques in the Methods section.*
☒	☐	A description of all covariates tested
☒	☐	A description of any assumptions or corrections, such as tests of normality and adjustment for multiple comparisons
☐	☒	A full description of the statistical parameters including central tendency (e.g. means) or other basic estimates (e.g. regression coefficient) AND variation (e.g. standard deviation) or associated estimates of uncertainty (e.g. confidence intervals)
☐	☒	For null hypothesis testing, the test statistic (e.g. F, t, r) with confidence intervals, effect sizes, degrees of freedom and P value noted<br>*Give P values as exact values whenever suitable.*
☒	☐	For Bayesian analysis, information on the choice of priors and Markov chain Monte Carlo settings
☒	☐	For hierarchical and complex designs, identification of the appropriate level for tests and full reporting of outcomes
☒	☐	Estimates of effect sizes (e.g. Cohen's d, Pearson's r), indicating how they were calculated

Our web collection on statistics for biologists contains articles on many of the points above.

Software and code

Policy information about availability of computer code

Data collection	Participant data was collected using REDCap version 11.1.29
Data analysis	Statistics and fitting were performed using MATLAB v.2019b, and GraphPad Prism version 10. MATLAB v.2019b scripts are available on GitHub (https://github.com/sigallab/NatureMarch2021). Sequences were analysed using the Standford Corona Virus Antiviral and Resistance Database (https://covdb.stanford.edu/sierra/sars2/by-sequences/) with HTML as output. The phylogenetic workflow is available at github.com/neherlab/BA286. The repository contains a specific list of sites (config/mask.tsv) that are masked in individual sequences. An interactive version of the phylogenetic tree is available at https://nextstrain.org/groups/neherlab/ncov/BA.2.86. Code is also available at Zenodo, https://doi.org/10.5281/zenodo.10082570.

For manuscripts utilizing custom algorithms or software that are central to the research but not yet described in published literature, software must be made available to editors and reviewers. We strongly encourage code deposition in a community repository (e.g. GitHub). See the Nature Portfolio guidelines for submitting code & software for further information.

Data

Policy information about availability of data

All manuscripts must include a data availability statement. This statement should provide the following information, where applicable:

- Accession codes, unique identifiers, or web links for publicly available datasets
- A description of any restrictions on data availability
- For clinical datasets or third party data, please ensure that the statement adheres to our policy

Viral isolates are available upon reasonable request. Source data are provided with this paper. Sequences of isolated SARS-CoV-2 used in this study have been deposited in GISAID and GenBank with accession numbers.

Ancestral variant with D614G substitution (B.1 lineage), GISAID: EPI_ISL_602626.1, Genbank: OP090658, https://www.ncbi.nlm.nih.gov/nuccore/OP090658.
XBB.1.5, GISAID: EPI_ISL_17506815, Genbank: OR782922, https://www.ncbi.nlm.nih.gov/nuccore/OR782922.

BA.2.86, GISAID: EPI_ISL_18226980, Genbank: OR775659, https://www.ncbi.nlm.nih.gov/nuccore/OR775659.

BA.1, GISAID: EPI_ISL_7886688, Genbank: OP090659, https://www.ncbi.nlm.nih.gov/nuccore/OP090659.1/.

All genome sequences and associated metadata in this dataset are published in GISAID's EpiCoV database (GISAID Identifier: EPI_SET_231003fr). To view the contributors of each individual sequence with details such as accession number, Virus name, Collection, Originating Lab and Submitting Lab and the list of Authors, visit 10.55876/gis8.231003fr. EPI_SET_231003fr is composed of 625 individual genome sequences. The collection dates range from 2021-12-14 to 2023-09-27; Data were collected in 48 countries and territories; All sequences in this dataset are compared relative to hCoV-19/Wuhan/WIV04/2019 (WIV04), the official reference sequence employed by GISAID (EPI_ISL_402124). For more information https://gisaid.org/WIV04

Research involving human participants, their data, or biological material

Policy information about studies with human participants or human data. See also policy information about sex, gender (identity/presentation), and sexual orientation and race, ethnicity and racism.

Reporting on sex and gender	Sex and gender was not considered in the study design, how this is information is collected and is based on self report by participants. Due to the nature of the pandemic, we did not select for sex/gender and used participant samples that were available at the time of experiments that were within the inclusion criteria.
Reporting on race, ethnicity, or other socially relevant groupings	Participants enrolled in the study were based on patient populations presenting to hospitals and clinics in KwaZulu-Natal, Durban, South Africa.
Population characteristics	Participant characteristics are included in supplementary tables S1-S8
Recruitment	for SARS-CoV-2 infected participants, blood samples were obtained from adults with PCR-confirmed SARS-CoV-2 infection who were enrolled in a prospective cohort study approved by the Biomedical Research Ethics Committee at the University of KwaZulu-Natal. Investigators were blinded to participant information. Blood samples used in the neutralization experiments were obtained under the same ethics. Samples were collected from 21 participants with Omicron XBB derived infection (13 female, 8 male), age ranges from 28-83. For samples used in the serosurvey analysis, samples were collected from 40 participants (33 female, 7 male) age ranges from 18-61. For the pre-Omicron vaccinated participants, samples were obtained from 19 participants (12 female, 7 males) age ranges from 22-75. For the BA.1 infected participants, samples were collected from 19 individuals (14 female, 5 male) age ranges 26-81.
Ethics oversight	The Biomedical Research Ethics Committee at the University of KwaZulu-Natal approved the prospective cohort study (reference BREC/00001275/2020). The Omicron/BA.I and BA.2.86 was isolated from a residual swab sample with SARS-CoV-2 isolation from the sample approved by the University of the Witwatersrand Human Research Ethics Committee (HREC) (ref.M210752). The sample to isolate XBB.1.5 was collected after written informed consent as part of the COVID-19 transmission and natural history in KwaZulu-Natal, South Africa: Epidemiological Investigation to Guide Prevention and Clinical Care in the Centre for the AIDS Programme of Research in South Africa (CAPRISA) study and approved by the Biomedical Research Ethics Committee at the University of KwaZulu-Natal (reference BREC/00001195/2020, BREC/00003106/2021).

Note that full information on the approval of the study protocol must also be provided in the manuscript.

Field-specific reporting

Please select the one below that is the best fit for your research. If you are not sure, read the appropriate sections before making your selection.

[x] Life sciences [ ] Behavioural & social sciences [ ] Ecological, evolutionary & environmental sciences

For a reference copy of the document with all sections, see nature.com/documents/nr-reporting-summary-flat.pdf

Life sciences study design

All studies must disclose on these points even when the disclosure is negative.

Sample size	Samples size was not predetermined. We used samples available that met the inclusion/exclusion criteria. Samples sizes were sufficient as samples allocated into groups based on infecting variant showed the same trend. The same applied to the serosurvey results.
Data exclusions	We did not exclude participants that met inclusion criteria
Replication	Repeated neutralization assays by using different participant plasma, this was performed in 3 independent experiments and results showed the same trend. Repeat testing performed for viral replication analysis and cytopathic effect in 2 independent experiments, successfully.
Randomization	Participants were allocated into groups based on infecting variant and at the time of serosurvey.
Blinding	The study is an observational cohort study, investigators were blinded to participant data during data collection. De-identified data was made available to investigators.

Reporting for specific materials, systems and methods

We require information from authors about some types of materials, experimental systems and methods used in many studies. Here, indicate whether each material, system or method listed is relevant to your study. If you are not sure if a list item applies to your research, read the appropriate section before selecting a response.

Materials & experimental systems

n/a	Involved in the study	
☐	☒	Antibodies
☐	☒	Eukaryotic cell lines
☒	☐	Palaeontology and archaeology
☒	☐	Animals and other organisms
☒	☐	Clinical data
☒	☐	Dual use research of concern
☒	☐	Plants

Methods

n/a	Involved in the study	
☒	☐	ChIP-seq
☒	☐	Flow cytometry
☒	☐	MRI-based neuroimaging

Antibodies

Antibodies used	For virus neutralization assays, foci were stained with a rabbit anti-spike monoclonal antibody (BS-R2B12, GenScript A02058) at 0.5 µg/mL and a secondary goat anti-rabbit HRP conjugated antibody (Abcam ab205718) was added at 1 µg/mL
Validation	BS-R2B12, GenScript A02058:<br>https://www.genscript.com/antibody/A02058-MonoRab_SARS_CoV_2_Spike_S1_Antibody_BS_R2B12_mAb_Rabbit.html<br>Abcam ab205718:<br>https://www.abcam.com/products/secondary-antibodies/goat-rabbit-igg-hl-hrp-ab205718.html

Eukaryotic cell lines

Policy information about cell lines and Sex and Gender in Research

Cell line source(s)	The H1299-E3 (H1299-ACE2, clone E3) cell line was derived from H1299 as described in our previous work. H1299 was a gift from M. Oren, originally obtained from ATCC, (CRL-5803). The VeroE6 cells expressing TMPRSS2 and ACE2 (VeroE6-TMPRSS2), originally BEI Resources, NR-54970.
Authentication	Cell lines have not been authenticated.
Mycoplasma contamination	The cell lines have been tested for mycoplasma contamination and are mycoplasma negative.
Commonly misidentified lines (See ICLAC register)	None

Plants

Seed stocks	*Report on the source of all seed stocks or other plant material used. If applicable, state the seed stock centre and catalogue number. If plant specimens were collected from the field, describe the collection location, date and sampling procedures.*
Novel plant genotypes	*Describe the methods by which all novel plant genotypes were produced. This includes those generated by transgenic approaches, gene editing, chemical/radiation-based mutagenesis and hybridization. For transgenic lines, describe the transformation method, the number of independent lines analyzed and the generation upon which experiments were performed. For gene-edited lines, describe the editor used, the endogenous sequence targeted for editing, the targeting guide RNA sequence (if applicable) and how the editor was applied.*
Authentication	*Describe any authentication procedures for each seed stock used or novel genotype generated. Describe any experiments used to assess the effect of a mutation and, where applicable, how potential secondary effects (e.g. second site T-DNA insertions, mosiacism, off-target gene editing) were examined.*
