# Supplementary material for: Evolution and neutralization escape of the SARS-CoV-2 BA.2.86 subvariant
Source: Nat Commun. 2023 Dec 6;14:8078. doi: 10.1038/s41467-023-43703-3 (PMC10700484; doi:10.1038/s41467-023-43703-3)
Supplement: Supplementary file 2 — Peer Review File [file 41467_2023_43703_MOESM2_ESM.pdf]

nature portfolio

Peer Review File

Evolution and neutralization escape of the SARS-CoV-2 BA.2.86 subvariant

Open Access This file is licensed under a Creative Commons Attribution 4.0 International License, which permits use, sharing, adaptation, distribution and reproduction in any medium or format, as long as you give appropriate credit to the original author(s) and the source, provide a link to the Creative Commons license, and indicate if changes were made. In the cases where the authors are anonymous, such as is the case for the reports of anonymous peer reviewers, author attribution should be to 'Anonymous Referee' followed by a clear attribution to the source work. The images or other third party material in this file are included in the article's Creative Commons license, unless indicated otherwise in a credit line to the material. If material is not included in the article's Creative Commons license and your intended use is not permitted by statutory regulation or exceeds the permitted use, you will need to obtain permission directly from the copyright holder. To view a copy of this license, visit http://creativecommons.org/licenses/by/4.0/.

Reviewers' Comments:

Reviewer #1:
Remarks to the Author:
Herein Khan et al. present the first characterisation of a primary isolate of BA.2.86.
Strengths of the study are observations using a primary clinical isolate that is genetically intact across of cohort of donor samples with known infection and vaccination history. Preliminary data is also presented on the growth characteristics in an engineered VeroE6 cell line.
Whilst very topical and using a genetically intact primary isolate, the major concerns I have with this work are as follows:

1- Breadth in neutralisation responses over time improves in cohorts. This is well known across many independent studies. To gain a snapshot of a variant like this (BA.2.86) and its ability to lower neutralisation titers versus one in circulation (XBB.1.5), recent samples with known vaccine and case histories are preferable. In review of the donor samples in tables S1 and S2, there are a sum total of 15 donors where the samples have been collected in 2023. Also, the justification of testing of samples from 2021 against BA.1 and BA.2.86 needs to be outlined in more detail. The majority of omicron lineages in that latter setting will have low neutralisation titers, so how this relates to the current state of the pandemic and the potential of this variant is unclear.
2- The donor mix is of interest as there is analysis of HIV +ve and -ve donors and these observations are important. That said, given the complexity of cohorts now with multiple layers of immunological experience, can comparisons be made when comparing groups with small numbers? The median ages, IQR of ages and male to female ratios would be good to be summarised here to see if one variable can be tested appropriately. For instance, in comparing groups (eg, HIV +ve vs HIV-ve) similar range in age and equally accounted for across male and female will be important in analysis of these small groups.
3- The virological characterisation of the isolate has limited resolution. The assays are well done and provide the first line of observations, but they simply represent the growth of primary isolates in a engineered VeroE6 cell line with a known defect in innate immunity. So whilst the growth is in line with a Omicron lineage like XBB.1.5 and not an early B clade (It would be good to know what actual clade this is and not have this listed as D614G like a pseudotyping study), its relative fitness against XBB.1.5 is completely unknown. In addition, its growth rate in a VeroE6 line would expected to be similar to another Omicron lineage in a cell line with limited antiviral restrictions.
4- Related to 3. Is the citing of reference 11, which is a pseudotyping study with only ACE2 expressed on the target cell. So comparing a primary isolate growing in cells that at least expresses both in vivo entry requirements (Ace2 and Tmprss2) would be good to outline here. The authors might wish to discuss another preprint that has been recently posted after submitting their work: https://www.biorxiv.org/content/10.1101/2023.09.11.557206v1
Here a cell line that is not engineered and closer to targets in the lung (Calu3) apparently favours BA.2.86 entry, albeit when using a pseudotype based assay.
Given this current submission is the only one using an actual primary isolate, it would be good to highlight this primary difference to these other works and if possible try and do a simple growth assay using the Calu3 cell or provide the isolate to teams that can do this experiment or alternatively this experiment in primary epithelia cultures if possible. The latter does take time, but infection of a Calu3 line can be done in a similar time frame to the data presented for the Vero line.

Minor points:
1- Reference 2 is a modelling study and it is unfortunate the large seminal studies that actually generated the data and this observation in clinical trials are not routinely cited over what is largely a review piece of their important work.
2- The use of a variant isolate that will compete with BA.2.86 both now and in the future. One would expect EG.5.1 or isolates with the 455 + 456 spike polymorphisms to be the competitors to this isolate in the community at present. It would also future proof this work by observing the responses to a current variant that may simply outgrow and supplant BA.2.86 over time. Whilst surveillance is also lagging in many countries, as present snapshot of the variants that we identified around the same time of this isolate would be informative. Eg. What % prevalence does BA.2.86 represent in the present mix.
3- Given the data observing lack of activity with Sotrovimab for BA.2.86 in pseudotyping assays,

data confirming this is the case in a primary isolate is indeed important, if BA.2.86 or related sub-lineages were to become dominant globally, then the use of this last monoclonal therapeutic may need to be revised in various therapeutic guidelines.

Reviewer #2:
Remarks to the Author:
In the present study Khan and coworkers describe the identification of a new SARS-CoV-2 variant BA.2.86 in South Africa. Similar variants were found in other places of South Africa and elsewhere. Based on the sequences of the studied strains and related sequences the authors speculate that the BA.2.86 variant was likely originating from South Africa. The authors also show that the BA.2.86 variant shows significant immunoevasion compared to ancestral D614G strain, but the level of immunoevasion is roughly equal to that seen by XBB.1.5 strain. In addition, the authors show that XBB.1 and BA.2.86 form smaller plaques i.e the spread of the viruses in cell cultures are significantly slower than that of the D614G virus. This is a short report that contains a limited amount of information i.e. the basic virological and immunological characterization of the novel BA.2.86 virus, but the data is important. There are some comments related to data presentation.
1. Figure 1, especially paper B is too small which does not allow the reader to see the details of phylogenetic analysis. At least the BA.2.86 portion should be enlarged.
2. In Fig. 1 and S1 the number of analysed serum specimens should be indicated in the figure legends even though the numbers can be calculated in enlarged figures.
3. It was not mentioned what virus dose (TCID50 amount) was used in plaque reduction neutralization assay.
4. In Table S1 the number of vaccine doses is not indicated. How many vaccine doses did each of the participant received?
5. Since the auhtors are taking a strong opinion that BA.2.86 is not more immunoevasive that XBB.1.5 it would be beneficial to provide more data with other BA.2.86 variant strains.

Reviewer #3:
Remarks to the Author:
Khan et al. present an engaging and intriguing manuscript that delves into the temporal and regional origins of the SARS-CoV-2 BA.2.86 Omicron subvariant. They also investigate the replication kinetics and potential for population immunity escape of BA.2.86, comparing earlier variants. The study effectively demonstrates that BA.2.86 may not be a significant cause for concern, as it exhibits in-vitro replication capacity and neutralization escape comparable to the cocirculating XBB.1.5 variant.

1. The assertion in the Introduction that a divergence of "30 mutations" in BA.2.86 from other Omicron subvariants is of utmost importance in a post-pandemic scenario with substantial global immunity lacks sufficient background context. The study cites the importance of neutralizing antibodies in protection from symptomatic infection but does not provide enough context regarding the significance of the observed antigenic differences, especially considering the prominent role of T cell immunity. Furthermore, the study weakens the assertion that BA.2.86 poses a major concern in terms of neutralizing antibody escape, as it does not show significantly more escape potential than co-circulating contemporary strains, despite having over 30 spike protein mutations.
2. A more extensive discussion regarding the potential origin of BA.2.86 is warranted. Consideration should be given to the differential sampling efforts in South Africa and Europe. Additionally, it would be valuable to explore the widespread polytomy in Europe associated with ORF1a: K1973R and discuss the timeframe within which BA.2.86 acquired over 30 mutations from the BA.2 basal polytomy, highlighting the rapid evolutionary capability of SARS-CoV-2 in the absence of significant population spread.
3. The authors show that in-vitro replication dynamics of BA.2.86 are similar to that of XBB.1.5. Regarding this inference, Figure 2C indicates that BA.2.86 may have a delayed but slightly higher replication capacity. While this difference may not be significant, the metric of fold change in CT values is a somewhat crude proxy for the amount of viable virus present. This conclusion would be more convincing if confirmed by an infectivity assay such as TCID50.

4. One major limitation of this study is the human serological cohort sample size. For example, Figure 1D suggests that BA.2.86 may escape neutralization by individuals infected with XBB family subvariants, but the number of participants is too few for a reliable inference.
5. Did the authors check for any differences in FRNT when the cohorts were stratified by vaccination type or timing of sample collection relative to infection/vaccination? Again, the small sample size may limit further stratification of the cohorts.
6. Line 69 states "emergence date in early May with an uncertainty of about two months." which could be slightly misleading. According to the nextstrain build, the uncertainty is two months before and two months after (Feb 20 – Jun 21), so four months in total.
7. Line 72 contains duplicate phrasing "GISAID accession".
8. Lines 101-102 – abbreviation is misplaced. "(CPE)" should follow the word "effect."
9. Citations on line 199 and 201 are formatted differently. Are they referring to the nextstrain build?
10. Figure 1 legend – the sentence "Numbers above columns…and fold change" is misplaced. Fold change is only indicated in panels E and F.
11. Figure 1A is cropped at the bottom.
12. Figure 1B legend should include details on how the tree was constructed. It is also somewhat difficult to differentiate the tip colors and to distinguish the node confidence intervals from the branches in some of the clusters. I suggest combining North and South America (as there is only one tip belonging to S America).
13. FRNT50 – abbreviation (focus reduction neutralization test) is not defined in text or figure legends.

23 October 2023

We thank the Reviewers for the insightful comments and we have now revised the manuscript to address them. Most importantly, we assembled: 1) a new plasma panel from individuals infected with XBB subvariants – we no longer include the older samples commented on by Reviewers 1 and 3 as having less relevance to the current state of immunity; 2) a completely new plasma panel from a serosurvey of participants who we enrolled after we received the Reviewer comments (September 2023). This panel shows the most recent state of neutralizing immunity in the South African population, which is surprisingly low against both BA.2.86 and XBB.1.5. These results greatly strengthen our conclusions, where we see little difference in escape from neutralizing immunity in BA.2.86 compared to XBB.1.5. Given the worrying low levels of neutralizing immunity to BA.2.86 which we found in the South African population, there may be a substantial number of BA.2.86 infections and it may evolve further.

We also experimentally determined that there is a strong linear correlation between qPCR Ct values and infectious viruses which shows that our replication comparisons between the viruses were valid as performed in the H1299-ACE2 cell line. H1299 cells, unlike Vero cells, are not considered interferon response deficient. We did not have the EG.5.1 or similar subvariant on hand for experiments but note that if BA.2.86 cannot outperform XBB.1.5, it is unlikely to outperform the more evolved EG.5.1. We have also expanded on the limitations, made the phylogenetic analysis clearer, and incorporated the other modifications as suggested by the Reviewers and detailed below.

Reviewer 1

1) To gain a snapshot of a variant like this (BA.2.86) and its ability to lower neutralisation titers versus one in circulation (XBB.1.5), recent samples with known vaccine and case histories are preferable. In review of the donor samples in Tables S1 and S2, there are a sum total of 15 donors where the samples have been collected in 2023. Also, the justification of testing of samples from 2021 against BA.1 and BA.2.86 needs to be outlined in more detail. The majority of omicron lineages in that latter setting will have low neutralisation titers, so how this relates to the current state of the pandemic and the potential of this variant is unclear.

To address this important comment, we did two sets of experiments:

First, we expanded our panel of participants with XBB derived subvariant infection to 21 participants. All are from 2023 (XBB derivative subvariant infections) except one participant who was confirmed XBB infected by sequencing. This panel replaces our previous panel of participants with BA.1 and later infections. This is now the new Figure 1C, and the associated participant description is in Tables S1 (summary) and S2 (per participant data).

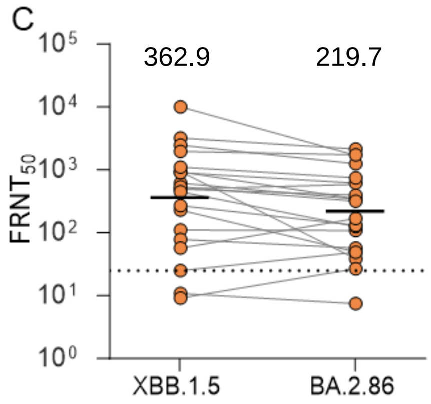

Second, we have assembled a plasma panel from a serosurvey we performed after we received the Reviewer comments and into which we enrolled 40 participants. While we don't know the date of infection for those participants, this panel represents a snapshot of the most recent neutralizing immunity in our region in South Africa. This is now the new Figure 1D, and the associated participant description is in Tables S3 and S4. The results showed surprisingly low levels of neutralizing immunity:

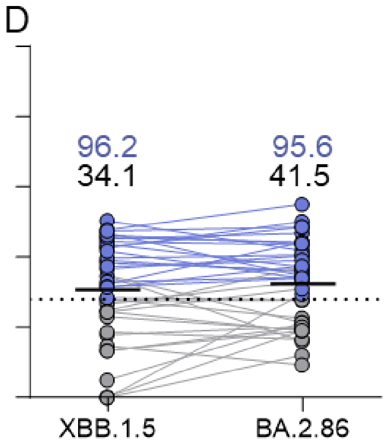

Here the black numbers above the points are $FRNT_{50}$ values for the entire group and the blue are the ones about the highest plasma concentration used (LOQ $FRNT_{50}$ = 25). In both cases, we did not observe significant differences between BA.2.86 and XBB.1.5.

2) given the complexity of cohorts now with multiple layers of immunological experience, can comparisons be made when comparing groups with small numbers? The median ages, IQR of ages and male to female ratios would be good to be summarised here to see if one variable can be tested appropriately. For instance, in comparing groups (eg, HIV +ve vs HIV-ve) similar range in age and equally accounted for across male and female will be important in analysis of these small groups.

This is an important comment. First, we added summary tables (Tables S1, S3, S5, S7). After consideration we think that stratification according to HIV status, vaccine status etc. is not helpful in the current immune environment. As the Reviewer notes, we now have multiple layers of immunological experience and many differences between people that may impact immunity. If we stratify fully, we will be comparing single participants. Therefore, we now present the data as neutralizing immunity for the South African population which has distinct features relative to other geographical locations such as less recent vaccination, no vaccines based on variant sequences, and higher HIV prevalence. We now state this on lines 146-151:

"Limitations to our conclusions include lack of stratification into subgroups based on age, HIV status, and detailed immune history. Given the current heterogeneity in immune histories, such stratification leads to comparison of very small groups. Our cohort is broadly representative of the South African population (41, 42) and our results should present the state of current neutralizing immunity in South Africa, which has distinct features relative to other locations such as less recent vaccination, no variant sequence vaccines, and higher HIV prevalence."

3) (Part 1) So whilst the growth is in line with a Omicron lineage like XBB.1.5 and not an early B clade (It would be good to know what actual clade this is and not have this listed as D614G like a pseudotyping study), its relative fitness against XBB.1.5 is completely unknown. In addition, its growth rate in a VeroE6 line would expected to be similar to another Omicron lineage in a cell line with limited antiviral restrictions.

We agree that viral growth in engineered cell lines may be a limitation. We now state this limitation on lines 164-165:

"The limitation of these results is that we used engineered cell lines, one of which was based on the Vero cell line which lacks an interferon response."

Because of this, we moved the replication data to the supplementary materials (Figure S5).

We specify the lineage of the ancestral SARS-CoV-2 as B.1 in the Table after line 367.

Regarding the replication experiment, it was done in the H1299 human epithelial cell line which is not known to be interferon deficient. We now emphasize this on lines 112-115:

"We then investigated whether there were any differences in infection properties in Vero-TMPRSS2 cells, a cell line known to lack an interferon response, and the H1299 human lung epithelial cell line stably expressing the ACE2 receptor, which has been reported to have an interferon response (28-32)."

We now explicitly state that we use the H1299 cells for the replication experiment on line 125:

"We also measured replication in H1299-ACE2 cells…"

(Part 2) Given this current submission is the only one using an actual primary isolate, it would be good to highlight this primary difference to these other works and if possible try and do a simple growth assay using the Calu3 cell or provide the isolate to teams that can do this experiment or alternatively this experiment in primary epithelia cultures if possible. The latter does take time, but infection of a Calu3 line can be done in a similar time frame to the data presented for the Vero line.

We agree with the Reviewer that measuring replication in Calu3 would be better, but these cells grow very slowly and we need a long lead time to expand the cultures to get enough cells to do the experiment and this is the same for our collaborators. Given the time sensitivity of the work we feel that the results in the H1299 cell line may be sufficient. As seen in a new Figure S6, XBB.1.5 has higher infectivity in these cells relative to ancestral SARS-CoV-2,unlike in Vero-TMPRSS2 cells, where ancestral is more infectious, consistent with what the Reviewer says about the role of the interferon response:

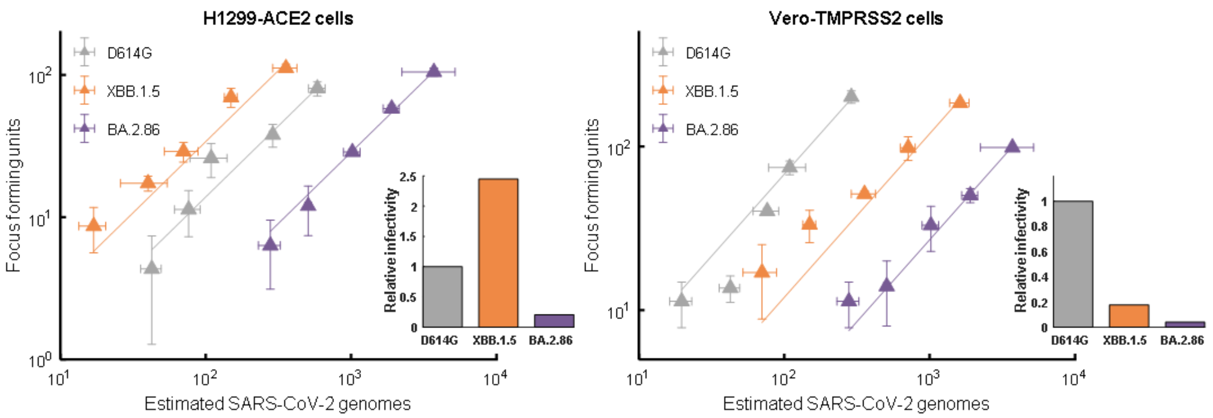

We made sure to acknowledge and reference the Calu3 results of the other group on lines 155-156:

"In contrast, another report showed increased BA.2.86 infectivity relative to XBB.1.5 in Calu3 cells (37)."

Given the importance of the interferon response for viral replication which the Reviewer points out, we repeated the analysis of focus area in H1299-ACE2 cells (Figure 2C):

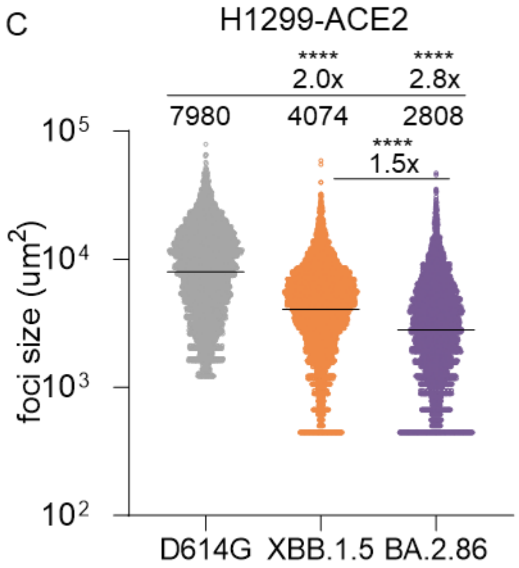

The differences in focus size in these cells between BA.2.86 and XBB.1.5 versus ancestral SARS-CoV-2 was smaller, consistent with increased evasion of the interferon response of Omicron subvariants narrowing the replication gap to ancestral SARS-CoV-2 (lines 159-163):

"Both BA.2.86 and XBB.1.5 made substantially smaller foci than ancestral SARS-CoV-2 foci. Interestingly, this difference was smaller in the H1299-ACE2 cell line, reported to have an interferon response. This is consistent with the notion that greater escape from the interferon response by Omicron subvariants (43) may offset other factors which make ancestral SARS-CoV-2 replicate faster."

Minor:

1) Reference 2 is a modelling study and it is unfortunate the large seminal studies that actually generated the data and this observation in clinical trials are not routinely cited over what is largely a review piece of their important work.

We have now added the references for the clinical trials alongside the Khoury et al. reference.

2) The use of a variant isolate that will compete with BA.2.86 both now and in the future. One would expect EG.5.1 or isolates with the 455 + 456 spike polymorphisms to be the competitors to this isolate in the community at present. It would also future proof this work by observing the responses to a current variant that may simply outgrow and supplant BA.2.86 over time. Whilst surveillance is also lagging in many countries, as present snapshot of the variants that we identified around the same time of this isolate would be informative. Eg. What % prevalence does BA.2.86 represent in the present mix.

We do not have an outgrowth of EG.5.1. We now state this limitation but note that if BA.2.86 cannot perform XBB.1.5, it is unlikely to outperform EG.5.1. This is discussed on lines 168-173:

“An additional limitation was that we compared BA.2.86 to XBB.1.5 and not to a more recent subvariant such as the Omicron XBB.1.9 derived EG.5.1. However, it would be expected that EG.5.1, having outcompeted XBB.1.5, should have advantages in immune escape from neutralization and/or replication over XBB.1.5. If BA.2.86 is not better at immune escape or replication relative to XBB.1.5, it would not be expected to gain these advantages over EG.5.1.”

We have added a new supplementary figure (Figure S2) which shows variant frequencies in South Africa including subvariant BA.2.86:

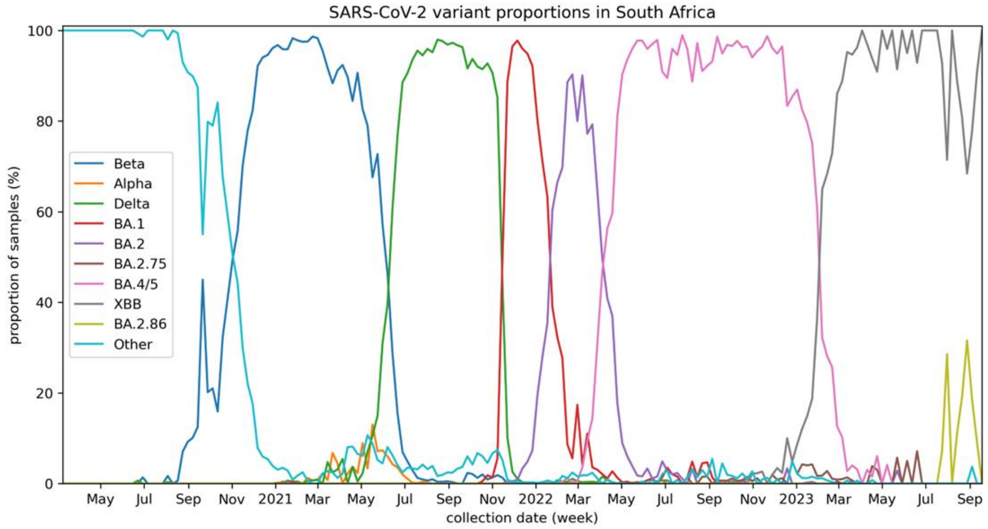

3) Given the data observing lack of activity with Sotrovimab for BA.2.86 in pseudotyping assays, data confirming this is the case in a primary isolate is indeed important, if BA.2.86 or related sub-lineages were to become dominant globally, then the use of this last monoclonal therapeutic may need to be revised in various therapeutic guidelines.

We do not have access to this antibody (neither do South African patients).

Reviewer 2

1) This is a short report that contains a limited amount of information i.e. the basic virological and immunological characterization of the novel BA.2.86 virus, but the data is important.

We thank the Reviewer for the support.

2) Figure 1, especially paper B is too small which does not allow the reader to see the details of phylogenetic analysis. At least the BA.2.86 portion should be enlarged.

We have now made Figure 1B clearer:

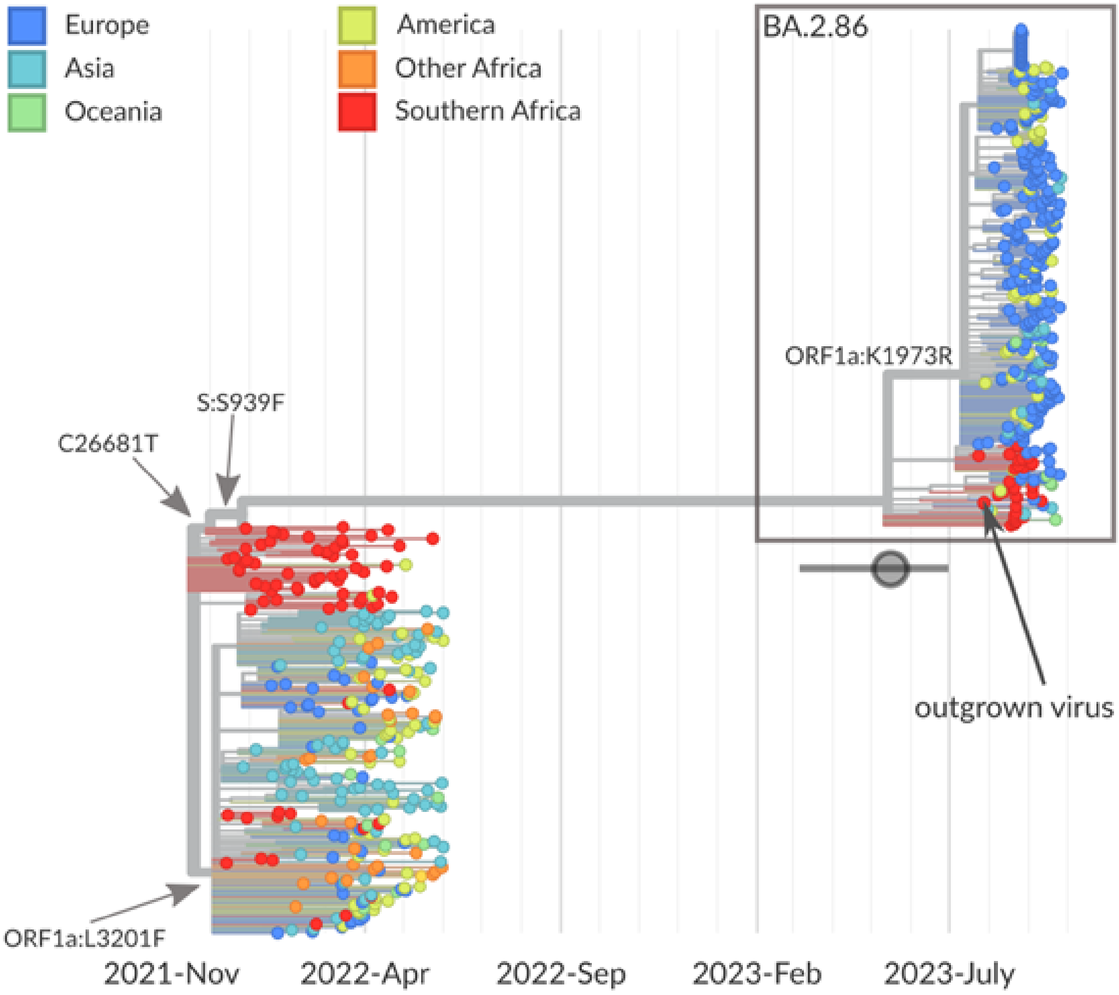

We have provided a new supplementary figure (Figure S1) with an enlarged phylogenetic tree around BA.2.86 spread:

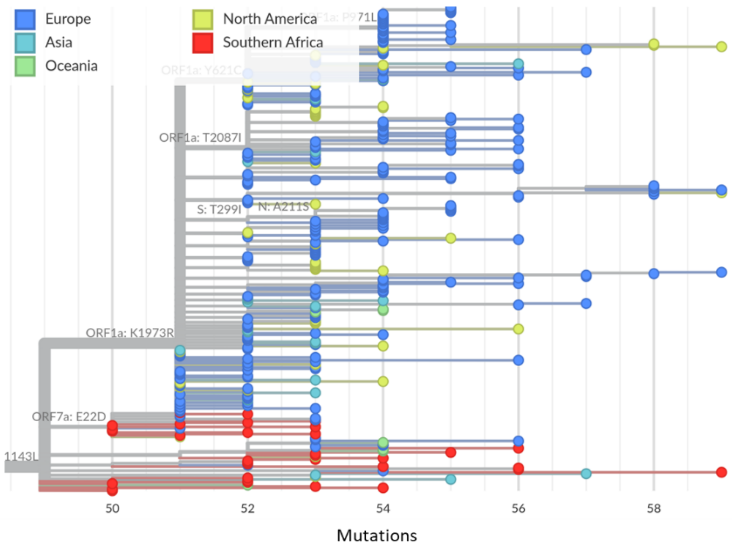

3) In Fig. 1 and S1 the number of analysed serum specimens should be indicated in the figure legends even though the numbers can be calculated in enlarged figures.

We appreciate the Reviewer pointing out this oversight. All neutralization panel figures now have the number of plasma samples used in the figure legends. We have also added the number of foci analyzed in Figure 2B and C.

4) It was not mentioned what virus dose (TCID50 amount) was used in plaque reduction neutralization assay.

We use focus forming units (FFU) to measure the number of infectious units. All focus reduction neutralization assays used an input of 100 FFU per 100 µL input (volume used to infect one well of a 96-well plate in the focus forming assay). This is now explicitly stated in the start of the focus forming assay description in the Materials and Methods (line 285):

"For all neutralization assays, viral input was 100 focus forming units per well of a 96-well plate."

5) In Table S1 the number of vaccine doses is not indicated. How many vaccine doses did each of the participant received?

The number of vaccine doses is now specified for all participants whose blood plasma is used in the neutralization panels in Tables S2, S4, S6, and S8.

6) Since the auhtors are taking a strong opinion that BA.2.86 is not more immunoevasive that XBB.1.5 it would be beneficial to provide more data with other BA.2.86 variant strains.

We would like to do this, however we do not have other isolates of BA.2.86 in-hand as outgrowth and specifically avoiding the in vitro R682W spike mutation has been more challenging than expected.

Reviewer 3

1) Khan et al. present an engaging and intriguing manuscript that delves into the temporal and regional origins of the SARS-CoV-2 BA.2.86 Omicron subvariant.

We thank the Reviewer for the support.

2) (Part 1) The study cites the importance of neutralizing antibodies in protection from symptomatic infection but does not provide enough context regarding the significance of the observed antigenic differences, especially considering the prominent role of T cell immunity.

We now add a discussion of the significance of the observed antigenic differences in the context of the role of T cell immunity, specifically that T cell immunity is more difficult to escape and therefore protection from severe disease should be maintained (lines 49-52):

"Protection from severe disease requires lower antibody levels (20), and also involves T cell responses (21-23) which tend to be conserved despite changes in the spike protein which characterize SARS-CoV-2 variants and subvariants (24, 25)."

(Part 2) Furthermore, the study weakens the assertion that BA.2.86 poses a major concern in terms of neutralizing antibody escape, as it does not show significantly more escape potential than co-circulating contemporary strains, despite having over 30 spike protein mutations.

We have now used similar wording to state our conclusions on lines 184185:
"Our data does not indicate that BA.2.86 is phenotypically very different from SARS-CoV-2 strains already in circulation."

3) A more extensive discussion regarding the potential origin of BA.2.86 is warranted. Consideration should be given to the differential sampling efforts in South Africa and Europe. Additionally, it would be valuable to explore the widespread polytomy in Europe associated with ORF1a: K1973R and discuss the timeframe within which BA.2.86 acquired over 30 mutations from the BA.2 basal polytomy, highlighting the rapid evolutionary capability of SARS-CoV-2 in the absence of significant population spread.

This is now done in a revised Figure 1B, and on lines 56-67 of the text:

"BA.2.86 shares the synonymous mutation C26681T and the spike substitution S939F with BA.2 genomes sampled in South Africa in early 2022, while it lacks the mutation C9866T (ORF1a:L3201F) that is present in the great majority of BA.2 sequences sampled outside of Southern Africa (Fig 1B).

Southern African sequences are also closely related to the putative ancestral sequence of BA.2.86. Most samples collected between mid-August and mid-September have 3-7 mutations relative to the most recent common ancestor (MRCA) of BA.2.86 (Fig S1). Of the 15 branches that emanate from the basal polytomy within BA.2.86, 11 are dominated by samples from Southern Africa (Fig 1B, Fig S1). Most sequences from the Northern Hemisphere fall into a second large polytomy designated as BA.2.86.1, separated from the basal polytomy by two mutations, including ORF1a:K1973R (Fig 1B). This sub-lineage has not been observed in South Africa at the time of analysis."

4) The authors show that in-vitro replication dynamics of BA.2.86 are similar to that of XBB.1.5. Regarding this inference, Figure 2C indicates that BA.2.86 may have a delayed but slightly higher replication capacity. While this difference may not be significant, the metric of fold change in CT values is a somewhat crude proxy for the amount of viable virus present. This conclusion would be more convincing if confirmed by an infectivity assay such as TCID50.

To address this important comment, we examined whether Ct values are a good indicator for viable viruses. We therefore used a dilution series and determined the relationship between Ct values (representing viral genome copies) to infectious viruses read out by focus forming units. We present the results in a new supplementary Figure 4:

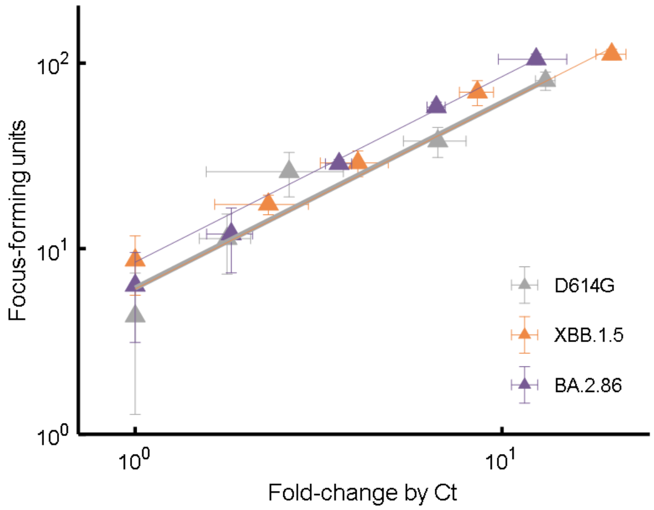

As can be seen by the linear fits shown as lines in the graph for each virus (all R^2>0.95), the fold-change in SARS-CoV-2 genomes as determined by Ct are predictive of the number of infectious viruses as determined by the focus forming assay.

We further examined the Reviewer's suggestion that BA.2.86 may have a delayed but slightly higher replication capacity by doing an additional independent repeat of the experiment and using the median instead of the mean as a measure for central tendency since the median is less affected by outlier values. The results (now Figure S5) shows that both BA.2.86 and XBB.1.5 overtake ancestral SARS-CoV-2 on day 4 but are very similar to each other:

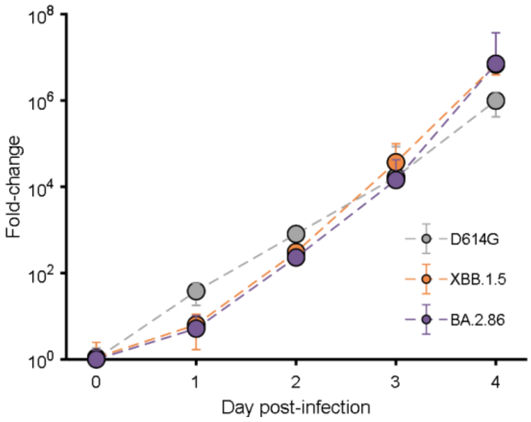

We also add more detail on how focus size reflects SARS-CoV-2 replication in a new supplementary figure (Figure S3):

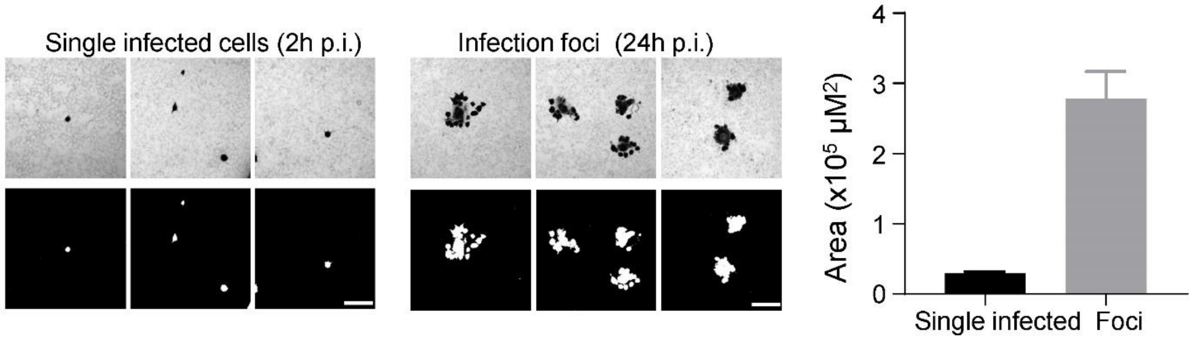

As can be seen from the zoomed in images of the foci, each is a mini-replication experiment which indicates viral spread to surrounding cells from a single infected cell over a defined time interval. Because of the large number of foci we can analyze, this should give us a more accurate readout of relative replication capacities.

In a new Figure 1C, we analyzed foci size 20 hours post-infection in the same H1299-ACE cells in which the viral replication experiments were performed (in addition to the foci in Vero-TMPRSS2 cells we previously analyzed):

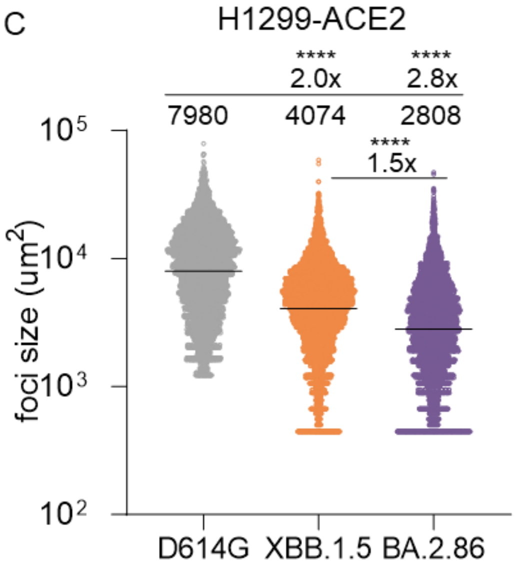

We found that foci sizes at that timepoint are smaller for both BA.2.86 and XBB.1.5 relative to ancestral SARS-CoV-2, with BA.2.86 foci being significantly smaller relative to XBB.1.5, consistent with the lower cell-free virus infectivity for BA.2.86 as shown in the response to point 3 of Reviewer 1.

5) One major limitation of this study is the human serological cohort sample size. For example, Figure 1D suggests that BA.2.86 may escape neutralization by individuals infected with XBB family subvariants, but the number of participants is too few for a reliable inference.

This was a limitation in the previous version which we addressed as described in the response to the first point of Reviewer 1. We have taken out the plasma samples from older Omicron infections and expanded our panel of plasma samples from XBB derived subvariant infected participants to n=21 plasma samples. We also included a serosurvey collected in September 2023 of plasma from South African participants (n=40 samples). We did not see a pattern where BA.2.86 shows escape in either of these groups. This is now presented as a new Figure 1C and D:

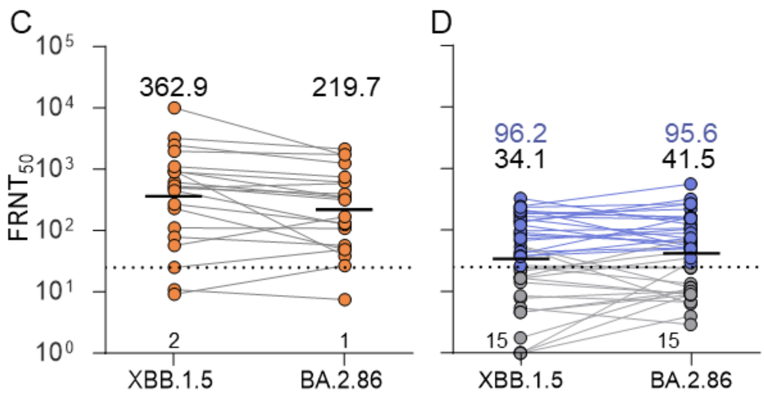

6) Did the authors check for any differences in FRNT when the cohorts were stratified by vaccination type or timing of sample collection relative to infection/vaccination? Again, the small sample size may limit further stratification of the cohorts.

We agree with the Reviewer that the small sample size limits our ability to stratify. Therefore, we now present the data as neutralizing immunity from the South African population which has distinct features relative to other geographical locations such as less recent vaccination, no vaccination with vaccines based on variant sequences, and higher HIV prevalence. We now state this on lines 146-151:

"Limitations to our conclusions include lack of stratification into subgroups based on age, HIV status, and detailed immune history. Given the current heterogeneity in immune histories, such stratification leads to comparison of very small groups. Our cohort is broadly representative of the South African population (41, 42) and our results should present the state of current neutralizing immunity in South Africa, which has distinct features relative to other locations such as less recent vaccination, no variant sequence vaccines, and higher HIV prevalence."

7) Line 69 states "emergence date in early May with an uncertainty of about two months." which could be slightly misleading. According to the nextstrain build, the uncertainty is two months before and two months after (Feb 20 – Jun 21), so four months in total.

We thank the Reviewer for pointing out the unclear description and we now fixed this in Figure 1B where we show the uncertainty interval (marked here with a red arrow):

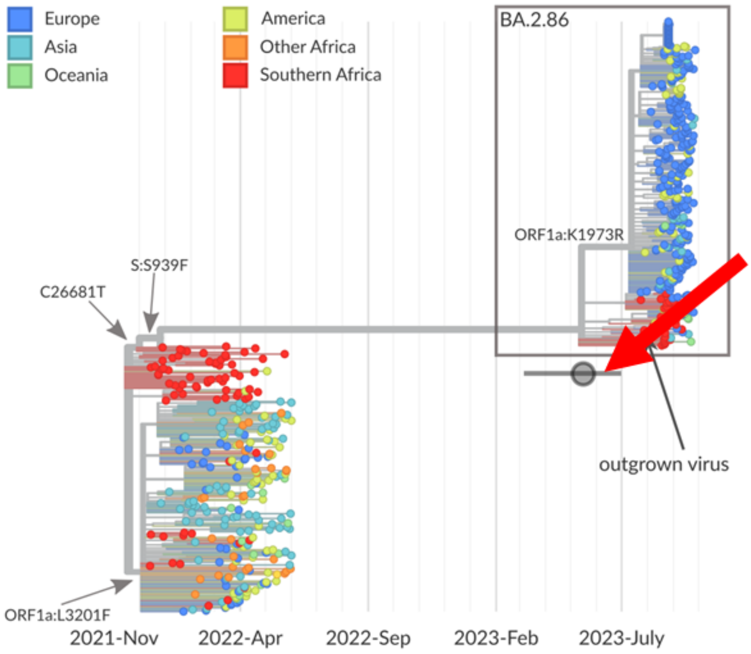

We now give a more precise description on lines 69-71 of the text:

"The estimate is corroborated by molecular clock analysis with TreeTime, which suggests an emergence date in mid-May with an uncertainty from early March to early July (Fig 1B)."

8) Line 72 contains duplicate phrasing "GISAID accession".

Fixed.

9) Lines 101-102 – abbreviation is misplaced. "(CPE)" should follow the word "effect."

Fixed.

10) Citations on line 199 and 201 are formatted differently. Are they referring to the nextstrain build?

This was an error – now reference 26.

11) Figure 1 legend – the sentence "Numbers above columns…and fold change" is misplaced. Fold change is only indicated in panels E and F.

Fixed.

12) Figure 1A is cropped at the bottom.

Fixed.

13) Figure 1B legend should include details on how the tree was constructed. It is also somewhat difficult to differentiate the tip colors and to distinguish the node confidence intervals from the branches in some of the clusters. I suggest combining North and South America (as there is only one tip belonging to S America).

As the Reviewer suggests, we merged North and South America to America in Figure 1B and made the Figure clearer.

The legend to Figure 1B now reads:

"Phylogenetic analysis. BA.2.86 sequences form a distinct cluster separated from BA.2 sequences circulating late 2021/early 2022 by a long branch. Outgrown sample marked by arrow. The BA.2.86 branch connects to samples with the mutations C26681T and C24378T (Spike: S939F) but lacks C9866T (ORF1a:K1973R) present in most BA.2 sequences. The tree was inferred using IQ-Tree (19) and time-scaled using TreeTime (22), see Materials and Methods for details. The emergence date of BA.2.86 and its uncertainty is indicated by a circle with horizontal bar."

14) FRNT50 – abbreviation (focus reduction neutralization test) is not defined in text or figure legends.

This is now done on lines 91-93:

"To determine neutralization capacity of live virus, we calculated the focus reduction neutralization test ($FRNT_{50}$) value, which is the inverse of the plasma dilution required for 50% reduction in infection focus number."

Sincerely yours,

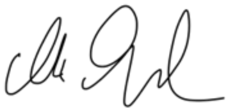

Alex Sigal, Africa Health Research Institute

Reviewers' Comments:

Reviewer #1:
Remarks to the Author:
The comments are primarily addressed in the revised manuscript.

My only remaining comment would be to tone down if possible the discussion on viral fitness in vitro. While well done, there are aspects of this that create confusion with respect to what the observations actually mean. For instance, the use of the H1299 Lung derived cell line is an important control and can (as the authors state) induce innate responses. That said, entry into the H1299 is identical in variants like Delta and early omicrons (https://www.ncbi.nlm.nih.gov/pmc/articles/PMC8942856/- See Figure Fig3 Panel A). The underlying reasons for this, is the H1299 poorly expresses TMPRSS2 and as such, the entry for all variants in this setting will be endocytosis. So is the innate response in this cell line a true reflection of what would occur with say a primary air-interface type 2 alveolar cell culture? Would that also be observed with the Calu3 cell line that does express TMPRSS2 at similar levels to primary type 2 cells?
Also on line 165 they refer to cell to cell spread of SARS CoV-2 being the main driver of infection and cell free SARS CoV-2 is of lower infectivity. The issue with this statement I see two-fold:
1- SARS CoV-2 spread globally has highlighted how effective cell free viral transmission can be.
2- Grown using the right cellular substrates and harvested within less than 24 hours can generate cell-free viral stocks that are very infectious on a per particle basis compared to other viral families.
I don't see this as a limitation and/or with enough datasets herein to really comment on this biology.

Overall the study is well done though and timely, with many important samples and controls to demonstrate where this variant sits within the neutralisation landscape at this point in time.

Given entry of SARS CoV-2 is complex and this complexity has increased upon the arrival of Omicron lineages, the change of a variants entry factor requirements needs to be mapped carefully and where possible tested in cellular substrates that would best provide observations of a change in tropism in vivo. The data for the assays is well done, but still remains preliminary & maybe stating "future studies that will test in primary cells and/or in in vivo animal models will give greater clarity with respect to evolving tropism of BA.2.86 and related sublineages"

Reviewer #2:
Remarks to the Author:
The authors have now adequately responded to all reviwers' comments.

Reviewer #3:
Remarks to the Author:
I am happy with the authors responses to the issues raised.

11 November 2023

Reviewer 1:

My only remaining comment would be to tone down if possible the discussion on viral fitness in vitro. While well done, there are aspects of this that create confusion with respect to what the observations actually mean. For instance, the use of the H1299 Lung derived cell line is an important control and can (as the authors state) induce innate responses. That said, entry into the H1299 is identical in variants like Delta and early omicrons (https://www.ncbi.nlm.nih.gov/pmc/articles/PMC8942856/- See Figure Fig3 Panel A). The underlying reasons for this, is the H1299 poorly expresses TMPRSS2 and as such, the entry for all variants in this setting will be endocytosis. So is the innate response in this cell line a true reflection of what would occur with say a primary air-interface type 2 alveolar cell culture? Would that also be observed with the Calu3 cell line that does express TMPRSS2 at similar levels to primary type 2 cells?

This is a valid comment and we have now addressed it by taking out most of the discussion regarding these results and emphasized the limitation of H1299 cells as being primarily SARS-CoV-2 infected through the endocytic pathway.

The summary of the results in the discussion now reads (lines 164-167):

“We did not observe substantial differences between BA.2.86 and XBB.1.5 in replication and spread between cells as detected by focus size, and no major differences were visible in cytopathic effect.”

The limitation of the H1299 system is further discussed and now reads (lines 173-180):

“A second limitation is that we used engineered cell lines which do not represent the full spectrum of virus-cell interactions: the Vero cell line which lacks an interferon response, and the H1299 cell line, having low levels of TMPRSS2 expression, has been shown to be predominantly infected by SARS-CoV-2 through the endocytic pathway (43) and therefore would not detect differences in infection through the TMPRSS2-dependent plasma membrane pathway. Therefore, our data is not inconsistent with another report showing increased BA.2.86 infectivity relative to XBB.1.5 in Calu3 cells which do use the TMPRSS2 pathway (37).”

Also on line 165 they refer to cell to cell spread of SARS CoV-2 being the main driver of infection and cell free SARS CoV-2 is of lower infectivity. The issue with this statement I see two-fold:
1- SARS CoV-2 spread globally has highlighted how effective cell free viral transmission can be.
2- Grown using the right cellular substrates and harvested within less than 24 hours can generate cell-free viral stocks that are very infectious on a per particle basis compared to other viral families.
I don't see this as a limitation and/or with enough datasets herein to really comment on this biology.

The discussion of cell-cell spread of SARS-CoV-2 has been removed.

Overall the study is well done though and timely, with many important samples and controls to demonstrate where this variant sits within the neutralisation landscape at this point in time.
Given entry of SARS CoV-2 is complex and this complexity has increased upon the arrival of Omicron lineages, the change of a variants entry factor requirements needs to be mapped carefully and where possible tested in cellular substrates that would best provide observations of a change in tropism in vivo. The data for the assays is well done, but still remains preliminary & maybe stating "future studies that will test in primary cells and/or in in vivo animal models will give greater clarity with respect to evolving tropism of BA.2.86 and related sublineages"

We agree and added the sentence on lines 181-182:

“Additional work in primary cells and animal models will give greater clarity on cell tropism, pathogenicity, and replication of BA.2.86 relative to other circulating strains.”

Sincerely yours,

Alex Sigal, Africa Health Research Institute
